# Genomic Modifiers of Natural Killer Cells, Immune Responsiveness and Lymphoid Tissue Remodeling Together Increase Host Resistance to Viral Infection

**DOI:** 10.1371/journal.ppat.1005419

**Published:** 2016-02-04

**Authors:** Alyssa Lundgren Gillespie, Jeffrey Teoh, Heather Lee, Jessica Prince, Michael D. Stadnisky, Monique Anderson, William Nash, Claudia Rival, Hairong Wei, Awndre Gamache, Charles R. Farber, Kenneth Tung, Michael G. Brown

**Affiliations:** 1 Department of Medicine, Division of Nephrology, University of Virginia, Charlottesville, Virginia, United States of America; 2 Beirne Carter Center for Immunology Research, University of Virginia, Charlottesville, Virginia, United States of America; 3 Department of Microbiology, Immunology and Cancer Biology, University of Virginia, Charlottesville, Virginia, United States of America; 4 Department of Biology, University of Virginia, Charlottesville, Virginia, United States of America; 5 Department of Pathology, University of Virginia, Charlottesville, Virginia, United States of America; 6 Department of Biochemistry and Molecular Genetics, University of Virginia, Charlottesville, Virginia, United States of America; 7 Department of Public Health Sciences, University of Virginia, Charlottesville, Virginia, United States of America; 8 Center for Public Health Genomics, University of Virginia, Charlottesville, Virginia, United States of America; La Jolla Institute for Allergy and Immunology, UNITED STATES

## Abstract

The MHC class I D^k^ molecule supplies vital host resistance during murine cytomegalovirus (MCMV) infection. Natural killer (NK) cells expressing the Ly49G2 inhibitory receptor, which specifically binds D^k^, are required to control viral spread. The extent of D^k^-dependent host resistance, however, differs significantly amongst related strains of mice, C57L and MA/My. As a result, we predicted that relatively small-effect modifier genetic loci might together shape immune cell features, NK cell reactivity, and the host immune response to MCMV. A robust D^k^-dependent genetic effect, however, has so far hindered attempts to identify additional host resistance factors. Thus, we applied genomic mapping strategies and multicolor flow cytometric analysis of immune cells in naive and virus-infected hosts to identify genetic modifiers of the host immune response to MCMV. We discovered and validated many quantitative trait loci (QTL); these were mapped to at least 19 positions on 16 chromosomes. Intriguingly, one newly discovered non-MHC locus (*Cmv5*) controlled splenic NK cell accrual, secondary lymphoid organ structure, and lymphoid follicle development during MCMV infection. We infer that *Cmv5* aids host resistance to MCMV infection by expanding NK cells needed to preserve and protect essential tissue structural elements, to enhance lymphoid remodeling and to increase viral clearance in spleen.

## Author Summary

Uncovering the genetic basis of resistance to viral infection and disease is critical to learning about how immune defenses might be adjusted, how to design better vaccines, and how to elicit effectual immune protection in human populations. Prior studies have shown that both MHC and non-MHC genes support host defenses, or endow specialized immune cells with efficient sensing or responsiveness to infection. Many additional resistance genes remain to be identified, including difficult to detect smaller-effect alleles, which might add to or interact with other genetic factors. Our grasp of the complex interaction involving these genetic elements is thus inadequate. We combined genomic and multiparameter phenotypic analyses to map and identify host genes that control immune cells or sensitivity to viral infection. We reasoned that some might also affect viral clearance. Thus we enumerated a range of immune cell traits in mice before and after infection, which permitted genomic analysis of viral immunity, and mapping of genetic modifiers for each trait. Our study demonstrates that distinct loci collectively regulate both NK cells and host resistance, which provides a framework to understand the genetic interactions, and a variety of potential novel targets to adjust NK cell functionality and host resistance to infection.

## Introduction

Yellow fever virus (YFV), once a major scourge of humanity, was one of the first viruses studied experimentally in mammalian hosts [1]. In pioneering studies, Nobel Laureate Max Theiler developed an inactivated YFV vaccine [2], and laid the groundwork for investigations into the genetic basis of host resistance to virus infection. Sawyer and Lloyd later observed that different strains of white mice are differently susceptible to YFV [3], and Lynch and Hughes solidified the point that YFV susceptibility is a heritable trait [4]. Many years later, the underlying cause of disease and effect of genetic variance on host resistance to viral infection and pathogenesis is still of vital interest [5], as it promises to reveal yet unknown molecular targets, signaling pathways and cellular networks with relevance to human health and disease.

Genetic analysis of host resistance to MCMV has been especially rewarding [6–8]. Recently discovered genes encode virus sensors and ligands, cytokines and receptors, signal transducers, and effector molecules that either increase or decrease host resistance to infection [9–16]. Often, these molecules are related to cellular immunity, including a clutch of polymorphic NK cell receptors that specifically bind ligands on virus-infected cells [17–24]. Yet, our understanding of the genetic influences on NK cells in the response to viral infection remains incomplete.

We established several mouse models to explore the effect of MHC class I (MHC I) polymorphism on NK cells in viral immunity [25]. MHC I D^k^ confers dominant MCMV resistance in MA/My and C57L-derived transgenic D^k^ mice, while D^b^-expression in C57L and MA/My-derived congenic M.H2^b^ mice does not [26]. The D^k^ resistance effect requires NK cells that express Ly49G2 (G2), an inhibitory receptor that binds D^k^ and protects against viral spread [24]. Thus, decreased D^k^ expression on infected cells might release the G2-specific brake on NK stimulatory signals, therefore helping to eliminate MCMV targets [25,27]. However, G2's precise role in host resistance is still under investigation.

While analyzing the MHC effect on NK cells, we found that D^k^-dependent MCMV resistance is greater in C57L-derived mice, relative to MA/My mice [26]. Thus, C57L genetic modifiers may increase host resistance to infection. However, modifier genetic loci have so far eluded detection, likely due to the prominent role of D^k^. Although forward and reverse genetics approaches have uncovered many pathogen resistance genes, neither strategy is ideally suited to resolve smaller genetic effects. Moreover, reverse genetics relies on introducing novel mutations that result in phenotypic abnormalities, so it is not a practical way to identify or characterize natural allele variants with distinct effects on immune function or pathogen resistance.

We thus set out to map and characterize variable genetic effects (quantitative trait loci, QTL) that shape immune features in naïve and MCMV-infected mice. We expected that virus-responsive immune cell traits and those that provide critical MCMV resistance would be revealed through common QTL linkages at defined chromosome positions across the genome. To guard against spurious results, we generated an additional cohort of MHC I D^k^-disparate offspring that were given lower dose MCMV infection. We found that defined genomic regions controlled either naïve or MCMV-responsive NK cell features, while others affected both sets of traits. While the MHC I D molecule has a central role in virus resistance and NK cell functionality, genetic dissection allowed many additional immune cell and host response traits to be separately mapped and verified. One such newly discovered non-MHC QTL remarkably controlled the integrity of the splenic environment, the prevalence of NK cells, and overall resistance to virus infection.

## Results

### Genomic profiling of MCMV immunity

Previously we assessed the immune response to MCMV with a deep phenomic approach [23]. The original high dose (HD) cohort [23] and a second cohort of MA/My x C57L hybrid mice given lower dose (LD) infection are included in the current analysis (**S1 Fig**). All mouse genome-wide genotypes were assessed and verified using automated SNP analysis on an Illumina platform. We performed genome scans to detect and map host resistance and immune response modifier QTL. Briefly, pre- and postinfection quantitative traits from both cohorts were separately analyzed using the quantitative genetic mapping package R/qtl in the statistical computing program R [28]. **Table 1**consolidates the data, conservatively reporting the most significant genetic intervals (QTL) detected. Results are ordered by chromosome, and then by position with overlapping genetic intervals for the same trait detected in both cohorts, or distinct traits detected in at least one cohort listed.

**Table 1:** Genome-wide scan results for immune and host response traits.

Trait^a^	Chr	LD Infection		HD Infection		QTL^c^	
		Pos (CI)^b^	LOD (pval)	Pos (CI)	LOD (pval)		Linked Loci^d^ (cM)
Body Weight (%Δ)	1			92.1 (63.3–96.1)	5.0**	*Vbwc-1*	*Tgfb2 (90)*
Body Weight (Post)	1			96.1 (84.9–96.1)	4.6**	*Vbwp-1*	*Traf5 (97)*
G2^neg^ (Pre %)	2	31.8 (27–39.3)	10.8****	26.8 (1.8–36.8)	14.2****	*Nktmg2n-2*	*Tnfaip6 (30)*
Body Weight (Post)	2			44.3 (8.3–56.4)	6.3***	*Vbwp-2*	*Prkra (45)*
Body Weight (%Δ)	2			50.6 (27.7–57.8)	4.1*	*Vbwc-2*	
Body Weight (Pre)	3			40.0 (35.4–68.7)	5.0**	*Mbw-3*	
Body Weight (Post)	3			62.0 (2.0–80.5)	4.7**	*Vbwp-3*	*Nfkb1 (63)*
G2^neg^ (Pre %)	3	62.0 (54.7–80.5)	5.3**	76.2 (2.9–80.5)	4.2*	*Nktmg2n-3*	*Ifi44l (76*.*9)*
MCMV (Spleen)	4			86.5 (58.5–86.5)	4.4*	*Cmv6*	*Tnfrsf9 (82)*
Body Weight (Post)	4			86.5 (3.5–86.5)	4.6**	*Vbwp-4*	*Tnfrsf14 (86)*
DN (Post %)	4			86.5 (48.5–86.5)	4.6**	*Nkrmdn-4*	*Tnfrsf4*, *Tnfrsf18 (86*.*7)*
Body Weight (Post)	6			41.7 (11.7–53.0)	4.1*	*Vbwp-6*	*Trh (41)*
I/U SP (Pre %)	6	61.7 (53.8–66.3)	10.8****	53.7 (41.7–78.2)	5.9***	*Nktmiusp-6*	*Cmv4 (62*.*5)*
I/U SP (Post MFI)	6	61.7 (53.8–78.2)	6.8***			*Nkrmiuf-6*	*Nkc-Klra cluster (63*.*4)*
I/U SP (Post %)	6	64.0 (61.7–65.8)	23.8****			*Nkrmiusp-6*	*Klra8 (Cmv1; 63*.*4)*
NK1.1^+^ (Post %)	6	66.7 (59.2–77.7)	11.2****			*Nkrmnk1*.*1–6*	
I/U SP (Pre MFI)	6	69.5 (66.8–71.7)	4.3*	46.9 (37.5–56.7)	5.5**	*Nktmiuf-6*	
G2^neg^ (Pre %)	7	3.7 (3.7–6.2)	4.6*	3.7 (3.7–10.2)	5.1**	*Nktmg2n-7*	*Ncr1*, *Peg3 (3*.*9)*
Body Weight (%Δ)	7			16.9 (8.7–38.7)	4.3*	*Vbwc-7*	*Tgfb1 (14)*
Body Weight (Post)	7			38.7 (18.7–51.4)	7.0****	*Vbwp-7*	*Tyrobp (17*.*5)*
Body Weight (Pre)	7			43.8 (33.9–51.4)	5.4**	*Mbw-7*	
Spleen Weight (Post)	8	70.8 (3.8–71.1)	4.6**			*Vswp-8*	*Irf8 (70)*
Body Weight (Post)	8			70.8 (63.3–71.1)	5.1**	*Vbwp-8*	*Foxl1 (70*.*3)*
Body Weight (Post)	9			37.5 (2.5–59.6)	6.6****	*Vbwp-9*	*Rora (37*.*5)*
Body Weight (%Δ)	9			39.9 (20.7–59.8)	3.8*	*Vbwc-9*	*Rab27a (40)*
Spleen Weight (Post)	9	42.5 (2.5–57.5)	4.6*			*Vswp-9*	*Mapk6 (42*.*3)*
G2^neg^ (Pre %)	10	14.9 (9.8–23.2)	5.4**	13.4 (3.4–23.2)	8.7****	*Nktmg2n-10*	*Ifngr1 (8*.*5)*, *Raet1e*
G2 SP (Pre %)	10	14.9 (9.8–23.2)	4.5*			*Nktmg2sp-10*	*Ptprk (15)*
NK1.1^+^ (Post %)	13	2.1 (2.1–64.7)	4.2*	NM		*Nkrmnk1*.*1–13*	
I/U^neg^ (Pre %)	13	37.1 (27.1–45.5)	9.3****			*Nktmiun-13*	
G2^neg^ (Pre %)	13			37.1 (31.6–64.7)	6.4****	*Nktmg2n-13*	
I/U SP (Pre %)	13	41 (27.1–45.5)	6.6***			*Nktmiusp-13*	*Erap1 (40)*
I/U^neg^ (Post %)	13	47.8 (37.1–64.7)	4.5*			*Nkrmiun-13*	
Nkp46^+^ (Post %)	13	64.1 (45.1–64.7)	4.2*			*Nkrmnkp46-13*	
G2 SP (Post MFI)	13	64.1 (45.5–64.7)	4.0*			*Nkrmg2spf-13*	*GzmA (64)*
G2^neg^ (Pre %)	17	17.8 (14.6–22.8)	16.2****	15.7 (7.8–17.8)	19.6****	*Nktmg2n-17*	
G2 SP (Pre MFI)	17	17.8 (14.6–21.1)	25.3****	17.8 (15.7–18.6)	11.4****	*Nktmg2spf-17*	
MCMV (Log Spleen)	17	17.8 (12.8–22.9)	18.0****	18.6 (17.8–19.2)	57.1****	*H2D*	*H2D (Cmv3)*
G2 SP (Pre %)	17	18.6 (14.6–22.8)	10.9****	15.7 (9.0–25.9)	5.9***	*Nktmg2sp-17*	
Body Weight (%Δ)	17			18.6 (17.8–19.2)	41.6****	*Vbwc-17*	
Body Weight (Post)	17			18.6 (17.8–19.2)	7.2****	*Vbwp-17*	
G2 SP (Post MFI)	17	19.2 (14.6–22.8)	35.4****	27.8 (15.7–29.7)	9.6****	*Nkrmg2spf-17*	
G2 SP (Post %)	17	19.3 (9.0–22.9)	9.8****	18.6 (15.7–19.2)	47.4****	*Nkrmg2sp-17*	
MCMV (Log Liver)	17	NM		18.6 (17.8–19.2)	30.8****	*H2D*?	
Lymphocytes (Post Abs)	17	NM		19.2 (17.8–25.9)	16.8****	*Lrm-17*	
Nkp46^+^ (Post %)	17	14.6 (11.8–21.1)	12.8****	22.9 (21.1–25.9)	16.9****	*Cmv5*	*Tnfrs21 (~19*.*7)*, *Pla2g7*, *Prph2 (22*.*9)*, *Trem1*, *Apobec2 (24)*
Body Weight (Post)	19			50.5 (19.1–52.0)	6.1***	*Vbwp-19*	*Adrb1 (52)*
Body Weight (Pre)	19			50.5 (40.6–52.0)	5.5***	*Mbw-19*	*Adra2a (49)*
I/U SP (Pre %)	X			32.5 (19.1–72.9)	5.8***	*Nktmiusp-X*	*Il13ra1 (20*.*5)*
G2 SP (Pre %)	X			34.8 (25.9–46.3)	4.0*	*Nktmg2sp-X*	*Cd40l (31*.*2)*
Body Weight (Post)	X	40.4 (24.1–54.1)	8.5****	40.4 (21–72.9)	11.6****	*Vbwp-X*	*Mageb2 (40*.*4)*, *Il2rg (44)*
Body Weight (Pre)	X	49.1 (32.5–55.2)	10.4****	29.6 (19.1–72.4)	12.3****	*Mbw-X*	*Cxcr3 (44*.*6)*,*Cmv2 (~56)*

^a^Preinfection (Pre), postinfection (post), and change (Δ) traits were measured as described in [23]. Briefly, Ly49G2^+^ I/U^neg^ (G2 SP), G2^neg^ I/U^+^ (I/U SP), G2^neg^, I/U^neg^, G2^neg^ I/U^neg^ (double negative, DN), NK1.1^+^, NKp46^+^ NK cell percentages and receptor median fluorescence intensity (MFI) were measured in flow cytometry. Genome mapping results for naïve total I/U^+^ and G2^+^ NK cell percentages were excluded due to high background linkages to most chromosomes. Traits that were not mapped (NM) are listed.

^b^Scanone analysis of traits performed in R/qtl using the Haley-Knott method with significance values based on 10,000 permutations. Peak chromosome (Chr) position (Pos) linkages within the indicated confidence interval (CI) for QTL with **LOD** score > 3.8 if detected in both LD and HD cohorts, or if similarly mapped with at least one other significant QTL in either cohort are shown(*, 0.05 ≤ *p* ≤ 0.01; **, 0.01 < *p* ≤ 0.001; ***, 0.001 < *p* ≤ 0.0001; ****, *p* ≤ 0.0001).

^c^QTL nomenclature: Mouse body weight (*Mbw*), MCMV-induced body weight post (*Vbwp*), MCMV-induced body weight change (*Vbwc*), MCMV-induced spleen weight post (*Vswp*), NK cell trait (preinfection) modifier (*Nktm*), NK cell response (postinfection) modifier (*Nkrm*), and Lymphocyte response modifier (*Lrm*) traits are listed by chromosome. For example, *Vbwp-1* indicates that MCMV-induced postinfection body weight mapped on Chr-1. NK cell trait nomenclature also includes a suffix corresponding to the affected trait as indicated in the first column. Thus, *Nktmg2sp-10* indicates a Chr-10 QTL in control of preinfection percentage of Ly49G2 SP NK cells, while *Nkrmiuf-6* indicates a chr-6 QTL in control of postinfection Ly49I/U median fluorescence intensity. Novel MCMV resistance/susceptibility loci (*Cmv*) in control of virus levels after infection are also listed.

^d^Published loci mapped close to QTL identified in Table 1 are listed and can be accessed through the National Center for Biotechnology Information (http://www.ncbi.nlm.nih.gov/projects/mapview/map_search.cgi?taxid=10090).

**Fig 1:**
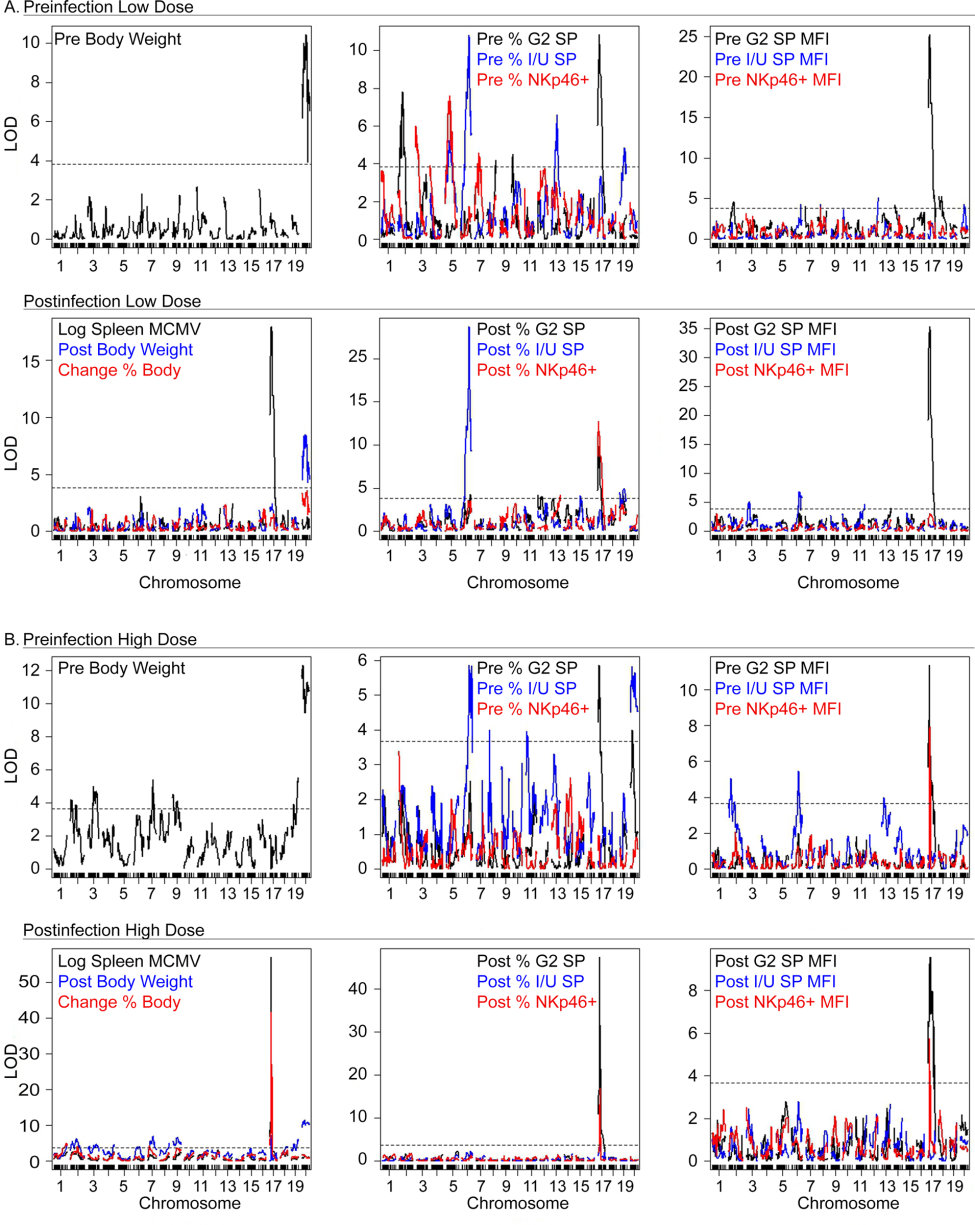
Genomic profiling of MCMV immunity. Multiple QTL chr maps for naïve and either LD (A) or HD (B) infected cohorts of mice. Shown are color-coded LOD profiles for the indicated pre- and postinfection traits for both cohorts.

As expected, sex-related naïve mouse body weight (*Mbw-X*) QTL mapped to overlapping genetic intervals on chr-X in both cohorts, but with slightly different peak logarithm of the odds (LOD) linkage positions, which relates the probability that an observed linkage was not due to chance (**Fig 1A and 1B, Table 1**). MCMV-induced body weight postinfection (*Vbwp-X*) QTL also mapped to this interval on chr-X, with near identical positions detected in LD and HD cohorts. As the only *Vbwp* QTL detected after LD infection, the results further suggested that MCMV-induced weight change was especially sensitive to chr-X weight control. Several smaller-effect mouse body weight QTL (*Mbw-3*, *Mbw-7*, *Mbw-19*) were also found and these coincided with postinfection weight control QTL (*Vbwp-3*, *Vbwp-7*, *Vbwc-7*, *Vbwp-19*) detected on chromosomes 3, 7 and 19, respectively (**Table 1**). Thus, several QTL were discovered that affected body weight in naive and MCMV-infected mice.

Host immune cell and response traits were likewise analyzed. Of the fifteen newly discovered NK cell trait modifier (*Nktm*) QTL, eight mapped respectively in both cohorts (**Table 1**). Twenty-four cohort-specific QTL were also identified **(Table 1)**, which suggested that subtle differences in infectious dose, cohort-size, or genetic make-up (**see S1 Fig**) could have revealed additional genetic associations. In general, broadly profiling genetically diverse mice with genome scans for immune traits provided a sensitive and reliable measure of the host response to viral infection, and led to the discovery of many novel QTL in control of immune cell homeostasis and reactivity.

As expected based on prior studies [20,25,29,30], a strong MCMV (log spleen) resistance QTL mapped to H-2*D* on chr-17 in both cohorts (**Fig 1A and 1B, Table 1**). No other single QTL was mapped with greater precision. Two critical MCMV-induced weight-control QTL (*Vbwp-17*, *Vbwc-17*) also mapped at the H-2*D* locus, but only in HD-infected mice (**Fig 1, Table 1**). Thus, these data demonstrated that H-2D controlled viral spread and the severity of weight loss (i.e. morbidity) solely after HD infection.

The prominence of D^k^ in host resistance led us to next assess its effect on NK cells in the response to MCMV. In fact, several NK cell modifier QTL in control of NKp46^+^ or G2^+^ NK cells and their response to MCMV were detected on chr-17. Interestingly, both NK cell trait (*Nktmg2sp-17*, *Nktmg2spf-17)* and response (*Nkrmg2sp-17*, *Nkrmg2spf-17)* modifiers of G2^+^ NK cells mapped to the H-2*D* locus. In contrast, QTL regulating Ly49I/U^+^ (I/U^+^) NK cells had no association to chr-17, and instead mapped to the NKC on chr-6 (**Fig 1A and 1B, Table 1**). Importantly, H-2D polymorphism specifically controls baseline G2^+^ NK cell features in naïve mice in addition to the G2^+^ NK response to MCMV, virus clearance and morbidity indices.

### Multiple NK cell and host response QTL together enhance host resistance to MCMV

To pursue genetic modifiers of MCMV immunity, we performed two-dimensional genome scans in R/qtl and analyzed each trait in possible two-locus QTL models. A higher LOD_av1_ value is indicative of an additive effect, without evidence of epistasis, while a higher LOD_int_ value is suggestive of genetic interaction. Two-locus modeling in R/qtl detected numerous QTL on a range of chromosomes with decidedly significant LOD_av1_ values, including many already discovered in Table 1. Thus, these QTL added to H-2*D*’s (pos 17.8 in Table 2) effect on MCMV burden or body weight, without evidence of interaction (**Table 2, S2A Fig**). Interestingly, seven common QTL positions precisely mapped on chromosomes 1, 3, 6 (pos 21.7, separate from the NKC), 11, 17 (pos 12.8, separate from the MHC), 18 and X added to both MCMV and body weight control, which suggested that a single shared QTL on each chromosome enhanced H-2*D*'s effect on both traits. Three additional QTL that mapped at similar positions on chromosomes 7, 8 and 12 also added to H-2*D*'s effect on MCMV and body weight control, which suggested these too mediate a shared locus effect. Five additional QTL on chromosomes 2, 9, 10, 13 and 19 had wider-ranging positions that added to H-2*D*'s control of either trait. Thus, we could not resolve whether common or distinct QTL on these chromosomes had a role. Nonetheless, both chr-13 (pos 37.1) and chr-19 (pos 49.1) QTL intervals that affected MCMV burden also overlapped with postinfection body weight intervals (**Table 2**). Several *Nktm* and *Nkrm* QTL mapped at the same chr-13 interval (**Tables 1 and 2**), further established strong support of at least one QTL on chr-13. While a chr-2 MCMV control QTL (pos 16.8) coincided with *Nktmg2n-2* and *Nktmg2sp-2*, a weight-control QTL (pos 31.8) corresponded better with two MCMV-induced weight control (*Vbwp-2*, *Vbwc-2*) QTL locations and the NK response modifier, *Nkrmdn-2* (**Tables 1 and 2**). Thus, two QTL were reliably detected on chr-2. Lastly, a chr-10 QTL for MCMV burden coincided with NK trait modifier QTL (*Nktmg2n-10*, *Nktmg2sp-10*), but a chr-10 (pos 13.4) weight control QTL could not be independently verified. In aggregate, the results implicated at least sixteen new QTL that regulate NK cells, virus clearance and morbidity indices, and most corroborated QTL reported in **Table 1**.

**Table 2:** Two-dimensional genome scan results for immune and host response traits.

**Evidence for additional QTL effects in MCMV immunity ^*a*^**							
**Trait**	**Chr1**	**Pos1**	**Chr2**	**Pos2**	**LOD_av1_**	**LOD_int_**	**Additional QTL^b^**
	1	92.1	17	17.8	9.02****	NS	
	2	16.8	17	17.8	8.50****	NS	
	3	77	17	17.8	3.28*	NS	
	4	73.5	17	17.8	5.30****	NS	
	5	17.5	17	17.8	4.03***	ND	
	6	21.7	17	17.8	3.48*	ND	
	7	18.7	17	17.8	3.26*	NS	
	8	63.8	17	17.8	3.17*	NS	
***MCMV (Log Spleen)***	9	57.5	17	17.8	5.38****	ND	
	10	13.4	17	17.8	3.97***	ND	
	11	62.7	17	17.8	5.80****	NS	
	12	50.6	17	17.8	3.92***	NS	
	13	37.1	17	17.8	3.95***	ND	
	17	12.8	17	17.8	13.3****	NS	
	17	17.8	18	12.4	5.82****	NS	
	17	17.8	19	49.1	3.99***	NS	
	17	17.8	X	19.1	4.51***	NS	
	1	92.1	17	17.8	11.42****	NS	
	2	31.8	17	17.8	8.55****	NS	
	3	77	17	17.8	4.73***	NS	
	6	21.7	17	17.8	4.77***	ND	
	7	13.7	17	17.8	6.74****	ND	
	8	58.8	17	17.8	3.89*	NS	
	9	27.5	17	17.8	8.92****	NS	
***Body Weight (%Δ)***	10	63.4	17	17.8	5.54****	NS	
	11	62.7	17	17.8	6.57****	NS	
	12	55.6	17	17.8	4.17***	NS	
	13	2.1	17	17.8	4.87***	NS	
	15	51.8	17	17.8	6.19****	NS	
	17	12.8	17	17.8	11.54****	NS	
	17	17.8	18	12.4	5.29****	NS	
	17	17.8	19	9.1	4.58***	ND	
	17	17.8	X	19.1	4.96***	ND	
	1	92.1	17	17.8	5.47****	NS	
	2	41.8	17	22.8	5.95****	NS	
	2	41.8	19	49.1	4.11***	ND	
	2	46.8	X	39.1	4.17***	NS	
	3	62	17	17.8	5.66****	NS	
	4	78.5	17	17.8	4.39***	NS	
	6	26.7	17	17.8	5.22****	NS	
	7	43.7	17	17.8	5.47****	NS	
	7	38.7	X	39.1	5.1****	NS	
	7	38.7	9	32.5	3.97***	NS	
***Body Weight (Post)***	8	68.8	17	17.8	5.07****	ND	
	9	37.5	17	17.8	6.33****	NS	
	9	37.5	X	59.1	4.29***	NS	
	13	37.1	17	17.8	3.89*	ND	
	15	26.8	17	17.8	3.81*	NS	
	17	12.8	17	17.8	7.91****	NS	
	17	17.8	18	42.4	3.91*	NS	
	17	17.8	19	49.1	5.79****	ND	
	17	22.8	X	29.1	7.75****	NS	
	19	49.1	X	39.1	4.54****	NS	
	1	87.1	17	17.8	4.98***	NS	*Nkrmdn-1*
	2	36.8	17	17.8	6.18****	NS	*Nkrmdn-2*
	3	77	17	17.8	4.42***	NS	
	4	33.5	17	17.8	5.16****	NS	
***DN (Post %)***	6	41.7	17	17.8	4.05***	NS	
	16	22.7	17	17.8	3.87***	NS	
	17	12.8	17	17.8	4.8***	ND	
	17	17.8	X	69.1	5.13****	ND	
	2	26.8	6	61.7	4.84***	NS	*Nktmg2sp-2 Nktmg2sp-6*
***G2 SP (Pre %)***	2	1.8	X	34.1	4.32***	NS	
	17	22.8	17	27.8	5.78****	NS	
	2	31.8	10	13.4	4.18*	NS	
***G2 SP (Pre %)*** ^***c***^	2	31.8	17	17.8	6.01***	NS	
	10	13.4	17	17.8	5.11***	NS	
	6	61.7	13	37.1	5.16****	NS	*Nktm-13*
	6	61.7	17	27.8	3.9*	NS	
***I/U SP (Pre %)*** ^***c***^	10	53.4	17	27.8	4.36***	NS	
	12	60.6	13	37.1	3.94*	NS	*Nktm-12*
	13	37.1	19	44.1	4.27***	NS	*Nktm-19*
	2	21.8	10	18.44	7.95****	NS	
	2	31.8	13	32.05	5.51****	NS	
	2	26.8	17	17.8	8.48****	NS	
	2	31.8	18	2.36	4.84****	NS	*Nktm-18*
	3	57	8	68.77	4.43***	NS	*Nktm-8*
***DN (Pre %)*** ^***c***^	8	68.8	10	13.44	4.96****	NS	
	8	68.8	13	37.05	5.85****	ND	
	8	63.8	17	7.8	5.38****	NS	
	10	13.4	13	37.05	5.57****	NS	
	10	18.4	17	7.8	7.95****	NS	
	13	37.1	17	17.8	5.89****	NS	
**Evidence for epistatic QTL interactions:**							
**Trait**	**Chr1**	**Pos1**	**Chr2**	**Pos2**	**LOD**_**av1**_	**LOD**_**int**_	
***MCMV (Log Spleen)***	9	47.5	X	29.1	ND	7.37***
	2	51.8	X	19.1	NS	6.23*	
***Body Weight (%Δ)***	3	57	14	48.1	ND	9.12****	
	4	78.5	12	50.6	NS	6.86*	
	5	72.5	X	39.1	ND	6.48*	
	6	61.7	17	17.8	4.2*	12.06****	
***G2 SP (Pre %)***	9	22.5	17	17.8	ND	6.88*	
	17	17.8	X	39.1	5.06****	8.06***	

^a^Scantwo analysis of traits performed in R/qtl using the Haley-Knott method with significance values based on 1000 permutations. Evidence for QTL on chromosomes (Chr1, Chr2) with peak linkage positions (Pos1, Pos2) with LOD_av1_ or LOD_int_ scores that exceeded 3.8, with *p* < 0.05 (0.05 < *p* ≤ 0.01*, 0.01 < *p* ≤ 0.001**, 0.001 < *p* ≤ 0.0001***, *p* ≤ 0.0001****) are shown. LOD_av1_ and LOD_int_ values that were not significant (NS) are listed, in addition to those not determined (ND) for several two-QTL models.

^b^Novel QTL detected in two-dimensional genome scans and not reported in Table 1.

^c^LD NK trait modifier QTL detected in scantwo analysis that substantiate QTL associated with MCMV burden or weight control.

Evidence of epistasis or interactive QTL (iQTL) pairs with significant LOD_int_ values that affected MCMV burden or weight control was also uncovered (**Table 2, S2B Fig**). Of these, all but the QTL on chr-5 and -14 could be independently verified in the genome scans of other traits. Interestingly, a chr-4 weight control iQTL that mapped similarly to *Cmv6*, coincided with an additive H-2*D* effect on MCMV burden (**Table 1**), in addition to its interaction with a chr-12 iQTL that further added to H-2*D*-dependent control of MCMV clearance and morbidity (**Table 2**). Thus, two-dimensional genome profiling uncovered and validated at least 18 novel QTL that affected viral spread or weight control during MCMV infection (**S2 Fig**). We infer that these genetic modifiers of NK cells and body weight together strongly influence overall host resistance to viral infection in both H-2D-dependent and independent ways.

### Combined effect of NKC and MHC polymorphism on NK cell reactivity and MCMV resistance

**Fig 2:**
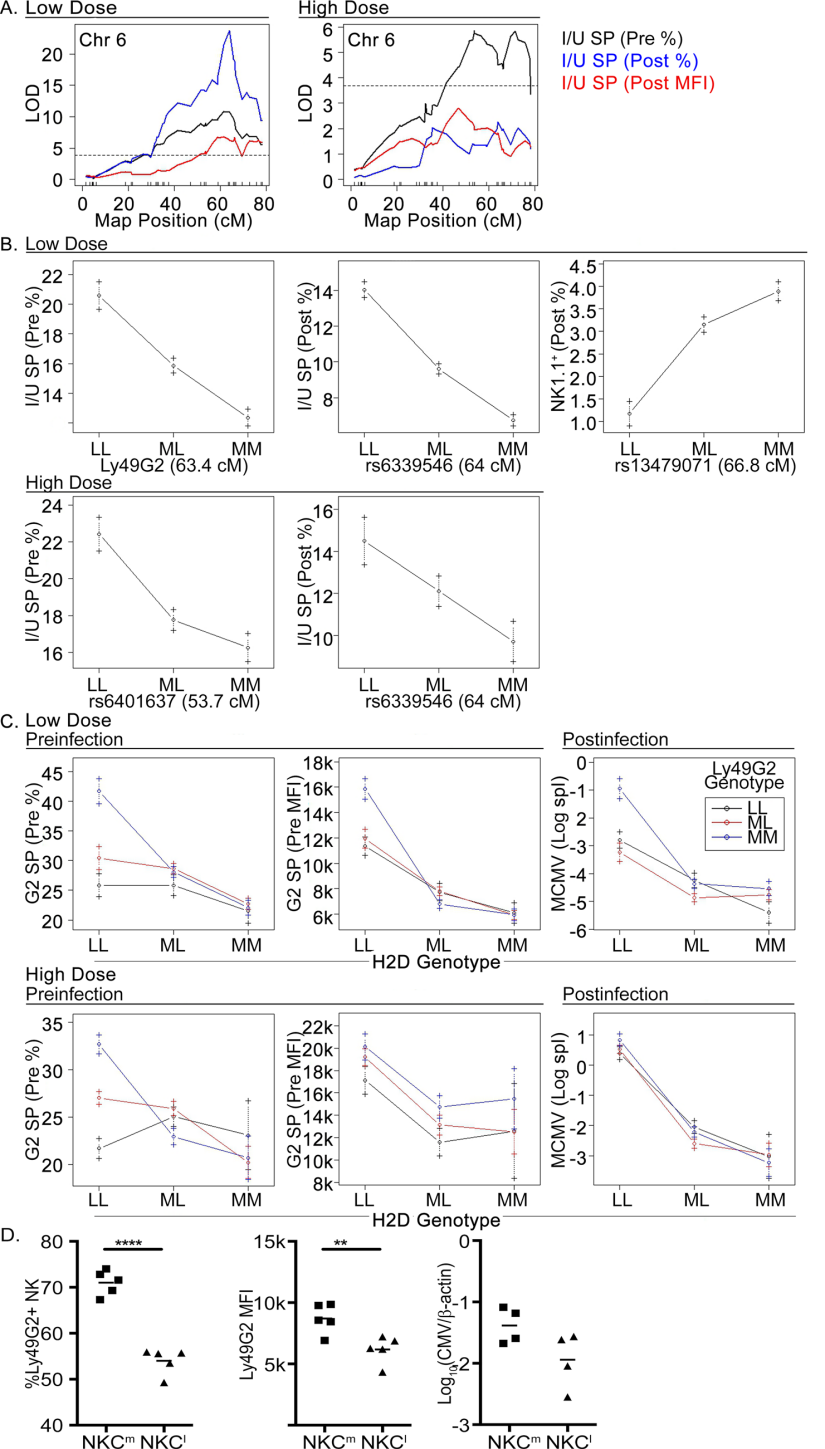
Combined effect of NKC and MHC polymorphism on NK cell reactivity and MCMV resistance. (A) Chr-6 interval mapping LOD plots for LD and HD mice. Shown are color-coded LOD profiles for I/U SP (i.e. G2^neg^) NK cell traits. (B and C) The plots show allele effects for defined traits for LD and HD mice with genotypes (L = C57L, M = MA/My, and ML = heterozygous) indicated for peak SNP marker positions. (D) The plots show splenic G2^+^ NK cell features for naïve C57L and C57L.M-NKC^m^ congenic mice, in addition to spleen MCMV burden after LD-infection. Data are representative of 3 independent experiments.

NKC-encoded receptor polymorphism has been shown to affect NK cells in the response to MCMV, and MHC-NK gene complex (NKC) epistasis contributes to MCMV control [20,30]. As expected, several modifier-QTL in control of NK cells, and I/U^+^ or G2^+^ NK subsets, were mapped to the NKC on chr-6 (**Tables 1 and 2; Fig 2A and 2B**). Chr-6, however, had no obvious effect on viral clearance or morbidity, so it was unlikely that Ly49 (Klra) polymorphism alone affected the variance in MCMV resistance in this genetic comparison. Nonetheless, genome profiling had uncovered a chr-6 QTL that added to H-2*D*’s effect on MCMV burden and weight control (**Table 2**). This result corresponds to a prior study showing that the C57L-derived NKC (NKC^l^) enhanced NK cell-mediated resistance after HD-infection in C57L mice without D^k^ [31]. Thus, we tested NK cell responsiveness by stratifying the phenotypic results by MHC and NKC genotypes. Interestingly, LD mice without D^k^ (i.e. H-2^b^ homozygous mice designated LL) had a lower viral burden if they also had a C57L-derived NKC (**Fig 2C**). A similar relationship was observed in HD mice, though not significant. While a single amino acid variation distinguishes G2 receptors in MA/My and C57L [24,32], the results suggested that G2 receptor polymorphism could have affected NK cell sensitivity to MCMV.

Further analysis of H-2^b^ homozygous offspring revealed that naïve G2-SP NK cells with C57L-derived G2^c57l^ receptors were fewer with lower G2 receptor MFI values, in comparison to mice solely expressing MA/My-derived G2^mamy^ receptors (**Fig 2C**). Whereas MHC I ligands of inhibitory NK receptors are known to affect NK cell and receptor expression features [33,34], the data suggested that the variant G2 receptors might differently bind H-2^b^ class I or class I-related molecules, which could have further resulted in MCMV resistance differences.

To corroborate our analysis of NK cells in H-2^b^ homozygous offspring, we next examined NKC congenic mice. As with the hybrid NK cells, naïve G2^+^ NK cell percentages and G2 MFI values were lower in NKC^c57l^ than NKC^mamy^ congenic mice (**Fig 2D**), which suggested that the disparate G2 receptors might also differently license NK cells. However, comparisons of NK cells in NKC congenic mice (**S3 Fig)**, or C57L and M.H2^b^ mice **(S4A Fig**) revealed similar sensitivity to *ex vivo* triggering via several different NK stimulatory receptors, and consequently no difference in NK cell licensing. Lower viral loads in NKC^c57l^ than in NKC^mamy^ spleens (**Fig 2C and 2D,** [31]), nonetheless prompted further analysis of G2^+^ NK cell reactivity to MCMV. In agreement with results from C57L-derived NKC congenic mice, lower G2^+^ NK cell percentages and G2 receptor MFI trends were observed in C57L mice, in comparison to MHC-matched, but NKC-disparate M.H2^b^ mice (**S4B Fig**). More intriguingly, host resistance to MCMV in C57L exceeded M.H2^b^, which directly corresponded to higher postinfection G2^+^ NK cell percentages and G2 receptor MFI (**S4B–S4D Fig**). C57L mice also exhibited significantly increased frequencies and numbers of total NK and G2^+^ NK cells producing greater amounts of IFN-γ (**S4E–S4G Fig**). D^k^-independent host resistance to MCMV therefore was also magnified in the C57L genetic background. We infer that G2^+^ NK cell phenotypes and effector functions are distinctly shaped by and sensitive to both NKC and non-NKC genetic effects, which further controls host-specific variations in virus response features.

### MHC-independent regulation of NK cell accrual after MCMV exposure

**Fig 3:**
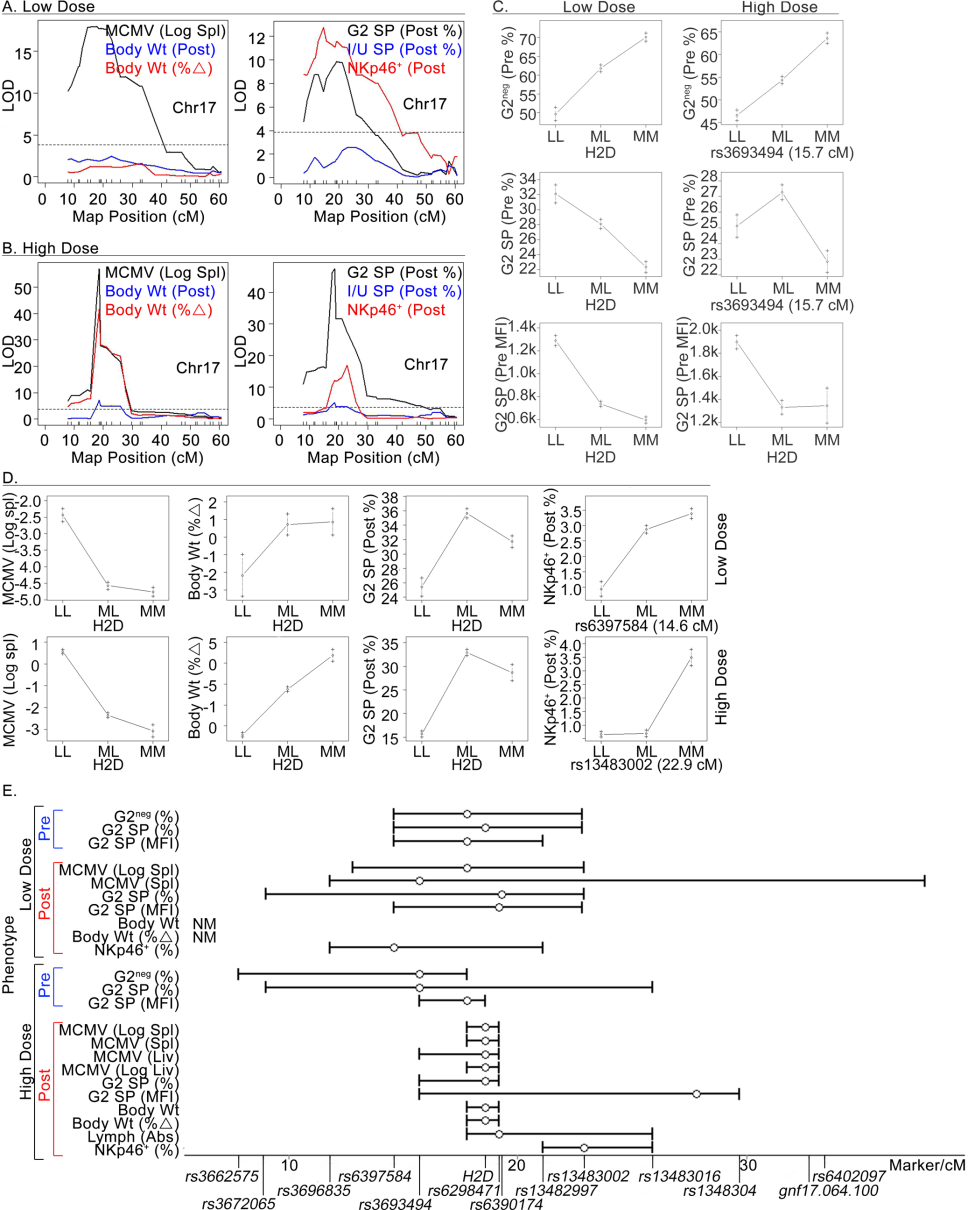
MHC-independent regulation of NK cell accrual after MCMV exposure. Color-coded chr-17 LOD plots for the indicated experimental traits in LD-infected (A) and HD-infected (B) cohorts of mice are shown. (C and D) Shown are allele effect plots for defined traits obtained for LD and HD mice with genotypes reported as in Fig 2. (E) A diagram of peak QTL positions (empty circles) with CIs (bracketed lines) for pre- and postinfection traits. A chr-17 physical map with defined marker loci and SNP positions is also shown. Two weight-related traits were not mapped (NM) in the LD cohort.

Given the robust D^k^ effect on NK cells and virus resistance, it was not surprising that the postinfection percentage of NKp46^+^ NK cells in spleen also mapped to chr-17 (**Table 1, Fig 3A and 3B**). Precision mapping in the HD cohort, however, resolved a peak QTL location (*rs13483002*) distal to H-2D (**Fig 3B**). The data implicated that a distinct locus outside of the MHC on chr-17 affected NK cell percentages after infection.

To address the question, we compared allele effect plots for several pre- and postinfection traits. We found that D^k^ had a profound effect on G2 receptor expression and NK cell subsets in naïve animals (**Fig 3C**). It also had a dominant effect on spleen MCMV burden after LD- or HD-infection (**Fig 3D**, compare H-2D genotype classes, LL and LM). More remarkable, D^k^ had a major impact on postinfection percentages of G2-SP NK cells and the severity of weight loss after infection (**Fig 3D**). In contrast, a C57L-derived locus (*rs13483002*) either dominantly prevented or failed to support NKp46^+^ NK cell accrual after HD-infection (**Fig 3D**, right panel). This effect was specific to NK cells since D^k^ affected the overall balance of total lymphocytes in the spleen (**Table 1, Fig 3E**).

A CI for the QTL controlling percentages of NKp46^+^ NK cells was mapped distal to D^k^ in the HD-infected cohort (**Fig 3E**). In support of this location, a very narrow R7-MHC congenic block (~300-kb D^k^-spanning segment [25]) was included in the genetic crosses used to generate the HD cohort so that many offspring had recombinant chromosomes between MHC I *D* and *rs13483002*. The results demonstrated that the D^k^-resistance factor alone was inadequate to support NK accumulation in spleen after HD-infection. Thus, at least two different chr-17 QTL control NK cells responding to MCMV.

### Verification of a non-MHC QTL on chr-17 that regulates NK cell accrual in spleen and host resistance to MCMV

**Fig 4:**
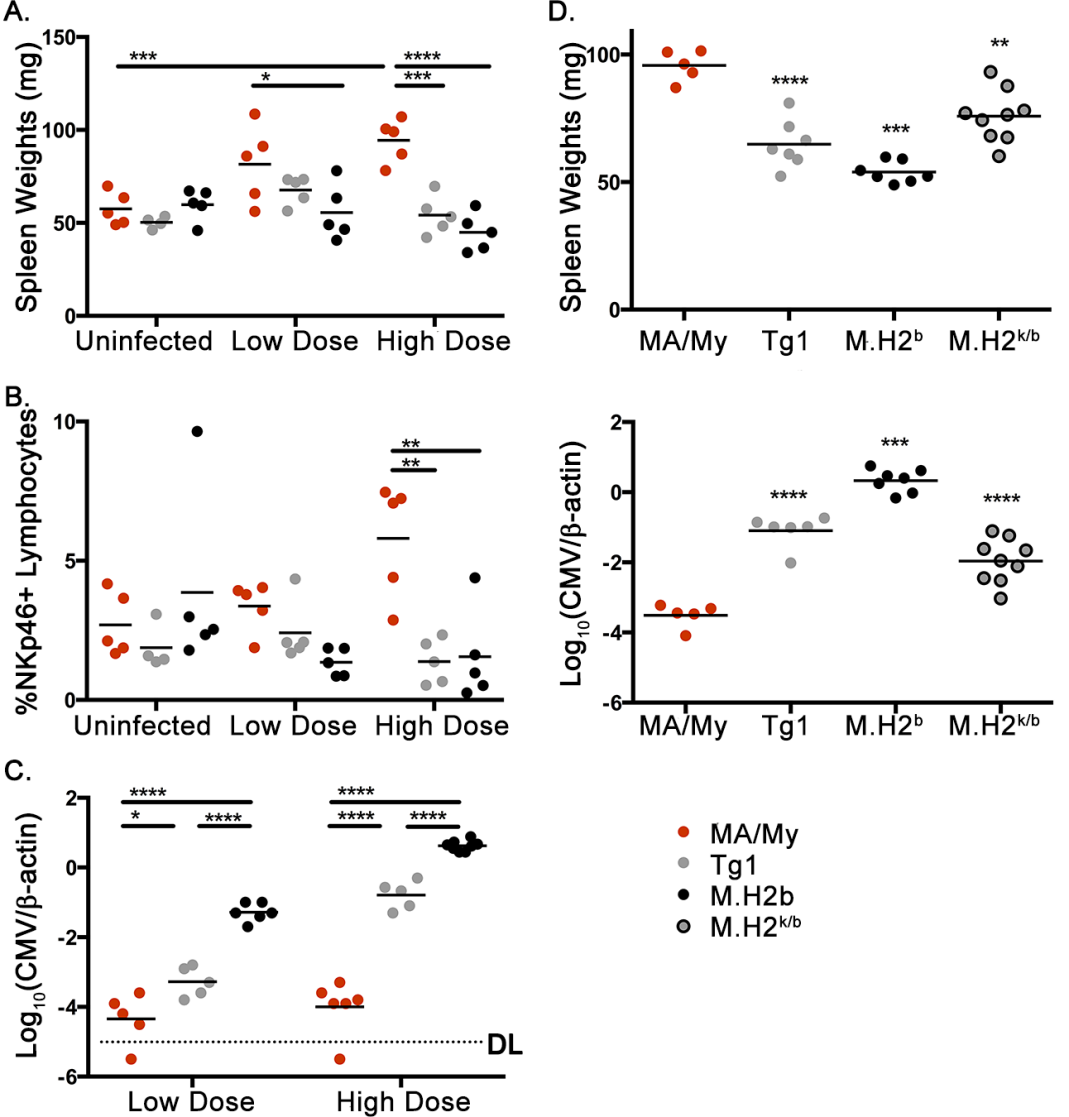
Verification of the *Cmv5* locus on chr-17 in MA/My-related congenic strains. (A and B) The plots show spleen weights (A) and percentages of NKp46^+^ NK cells (B) in uninfected, and LD- and HD-infected MA/My, M.H2^b^ and Tg1 (M.H2^b^ background) mice. The data are representative of 3 independent experiments. (C) The plot shows MCMV genome levels detected in spleen DNAs obtained from the indicated mice. The data are representative of 3 independent experiments. (D) The plots show spleen weights (left) and MCMV genome levels (right) for uninfected and HD-infected MA/My, M.H2^b^, Tg1 and (MA/My x M.H2^b^)F_1_ cross mice. The data are representative of 2 independent experiments.

To corroborate the *rs13483002* QTL effect, we compared the NK-cell response to infection in MA/My and two recently generated MA/My-derived strains, M.H2^b^ and M.H2^b^-TgD^k^ (hereafter referred to as Tg1), which expresses a genomic D^k^ transgene [25]. MA/My and Tg1 mice display MCMV resistance, whereas M.H2^b^ mice are highly susceptible to infection. Moreover, the M.H2^b^ congenic interval spans the *rs13483002* locus (for maps of R7 and M.H2^b^ congenic blocks, see Ref [23]). Overall NK cell percentages and numbers, in addition to spleen sizes, were equivalent in uninfected mice (**Fig 4A and 4B**). Within days after infection, however, MA/My spleens had grown much larger with significantly increased frequencies of NK1.1^+^ (**S5 Fig**) and NKp46^+^ NK cells (**Fig 4A and 4B**), which corresponded to lower viral loads (**Fig 4C**). In contrast, M.H2^b^ spleens failed to increase in size or frequency of NK cells after infection, regardless of viral dose, which resulted in greatly increased viral burden. Similar differences distinguished MA/My and Tg1 after HD-infection, regardless of enhanced viral control in Tg1 (compared to M.H2^b^ mice). Thus, D^k^ expression by itself is not sufficient to support accumulation of spleen NK cells in response to MCMV. Higher virus levels in Tg1 than MA/My mice further suggested that a C57L-derived susceptibility allele in the M.H2^b^ congenic interval is sufficient to decrease overall resistance. To test this, we next examined (MA/My x M.H2^b^)F_1_ cross mice after MCMV infection. Remarkably, M.H2^k/b^ spleens were smaller with significantly higher MCMV levels, in comparison to MA/My (**Fig 4D**). Thus, these data demonstrated that a C57L-derived susceptibility allele, provisionally designated *Cmv5* ^*s*^, interfered with the NK-mediated resistance effect of D^k^. Moreover, the results established that diminished control in M.H2^k/b^ is unlikely related to a D^k^ gene dose effect, since the same allele also interfered with host resistance in Tg1 mice with multiple integrated D^k^ gene copies and slightly higher protein expression [32]. Significantly higher percentages of NKp46^+^ NK cells and larger spleens in infected *Cmv5* ^*r*^ resistant MA/My mice provide added support of this interpretation. We infer that enhanced host resistance in MA/My due to increased accrual of NK cells is prevented by expression of at least one *Cmv5* ^*s*^ allele in more susceptible MA/My-derived (e.g. M.H2^b^) mice.

### *Cmv5* regulation of NK cell accrual in the infected spleen parallels its protection of secondary lymphoid organ structures

**Fig 5:**
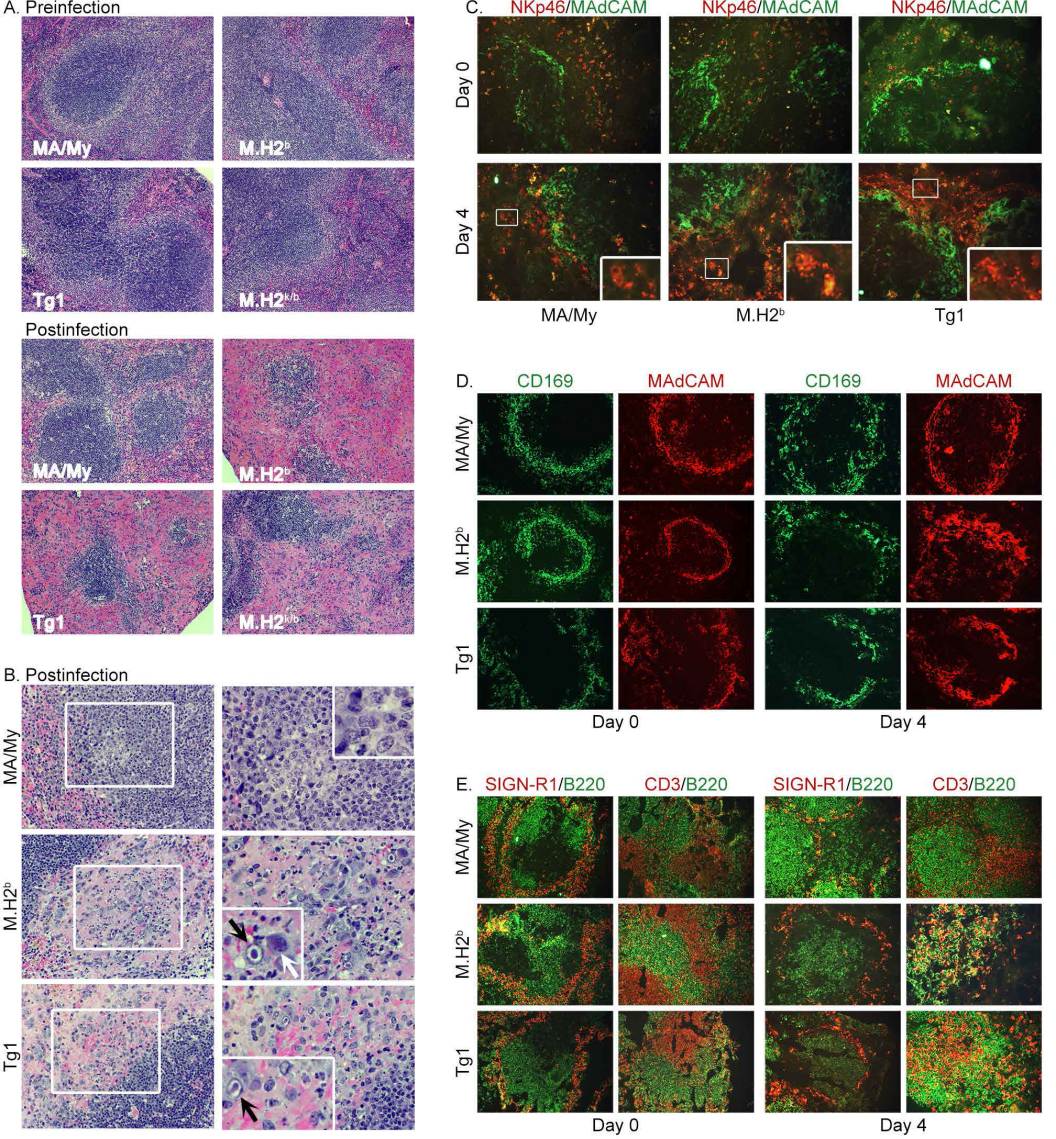
*Cmv5-*dependent protection of SLO structures and lymphoid remodeling in spleen during acute MCMV infection. (A and B) H&E-stained tissue sections obtained from uninfected and HD-infected (d 3.5) spleens of MA/My-related mice (magnification, (A) X100; (B) X200 or X400 with X800 insets). Images of infected spleen are representative of 4 to 8 mice per group in two independent experiments. Diminished SLO structural integrity is evident by comparison of WP and MZ regions, in addition to severe necrosis apparent in RP regions of spleens from the different strains. Pseudo-nodules of leukocytes heterogeneous in size and found near the MZ in MA/My mice frequently contained activated and mitotic figures inside square at low magnification on left, and at higher magnifications on right. Cytomegalic inclusions (black arrow) and plasmacytoid-like cells (white arrow) frequently detected in TgD^k^ and M.H2^b^ spleens are indicated (insets in right panels). (C-E) Frozen spleen sections stained with fluorescence-conjugated mAbs against NKp46^+^ NK cells and MAdCAM^+^ marginal sinus lining cells (magnification X200 with X600 insets) (C), MAdCAM^+^ or CD169^+^ MZ macrophages (magnification X100) (D), and both SIGN-R1^+^ MZ macrophages and B220^+^ B cells or CD3^+^ T cells and B220^+^ cells (magnification X100) (E, see text for interpretations).

Bekiaris et al. have previously shown that Ly49H^+^ NK cells are needed to protect spleen white pulp after MCMV infection [35]. Thus, we investigated whether NK cells similarly protected splenic architecture in *Cmv5*-disparate MA/My mice by comparing secondary lymphoid organ (SLO) structures after MCMV infection. Remarkably, within four days after infection, we observed decidedly ill-defined white pulp (WP) regions that were smaller and had less clearly delineated marginal zones (MZ) in M.H2^b^ and Tg1 mice, in comparison to infected MA/My or uninfected control mice (**Fig 5A**). Infected MA/My mice had more abundant lymphoid-like cells, in addition to pseudo-nodules of loosely packed mononuclear cells that were heterogeneous in size and shape, appeared to be activated with many mitotic figures, and located at the MZ at the edge of the WP (**Fig 5B**). In parallel, we observed severe necrosis in the expanded red pulp (RP), which was infiltrated with cells with viral inclusions, granulocytes, and large cells that resemble plasma cells in M.H2^b^ and Tg1 spleens, as compared to infected MA/My or uninfected controls (**Fig 5B**).

To validate the histological findings and *Cmv5* genetic regulation of SLO structure after MCMV infection, we next examined spleen sections for NK cells and SLO structural features by IF microscopy. As expected, we observed equivalent numbers of intact NKp46^+^ cells (ring-like staining patterns) in splenic RP of each strain before infection (**Fig 5C**). Afterward, NKp46^+^ NK cells were more closely associated with MAdCAM^+^ cells at the MZ (**Fig 5C**). In infected MA/My spleens, we observed intact NKp46^+^ cells, in addition to fragmented NKp46^+^ granular material, possibly representing NK cell debris. In contrast, intact NKp46^+^ cells were rare in infected M.H2^b^ and Tg1 spleens. Instead, extensive NKp46 staining of fragmented granular material, frequently concentrated in foci several times larger than an intact NK cell, was typical of infected M.H2^b^ and Tg1 spleens. These data suggested that necrotic NK cells and debris were much more prevalent in mice without *Cmv5* protection.

We also examined MAdCAM^+^ marginal sinus-lining cells and CD169^+^ macrophages at the MZ. At four days postinfection, MA/My spleens consistently had more discrete (rather than more diffuse) MZ staining patterns, than either M.H2^b^ or Tg1 (**Fig 5D**). Nonetheless, IF staining for T and B cells verified even more consistent differences and a selective loss of SLO structural features in M.H2^b^ and Tg1 mice after MCMV infection (**Fig 5E**). Prominently, in M.H2^b^ and Tg1 spleens, WP was marked by a lack of association between B220^+^ cells and the MZ. In addition, we observed disorganized regions of T and B cells, and a loss of B cell zone integrity. Together, these results establish that the M.H2^b^ congenic segment failed to protect spleen structures and NK cell accrual after infection. Related occurrences in Tg1 mice verified that D^k^ was inadequate to protect SLO structure after infection, even though it aided viral clearance compared to M.H2^b^ (**Fig 4**).

As the extent of viral control differs in MA/My and Tg1 spleens (**Fig 4**), we considered that apparent differences in splenic SLO integrity could be due to the variance in viral titers. In line with previous studies [23–25], we reasoned that G2^+^ NK-mediated resistance in MA/My and Tg1 is equivalent. Thus, we further assessed the effect of G2^+^ NK cells on SLO structure under both LD- and HD-infection conditions. As expected, G2^+^ NK cell depletion resulted in significantly diminished spleen weight, splenocyte numbers, and NK cell recovery after infection in both strains (**S6A and S6B Fig**). Virus levels were correspondingly higher in G2-depleted mice (**S6C Fig**). Thus, G2^+^ NK cells are critical. Nonetheless, residual MCMV resistance in G2-depleted MA/My still exceeded that observed in G2-depleted Tg1, which revealed that *Cmv5* exerted G2^+^ NK-independent effects on spleen and MCMV clearance (**S6C Fig**). Moreover, although spleen structure again waned with increased viral burden, G2-depleted MA/My spleens retained better defined WP and a higher density of small lymphocytes, in comparison to virus level-matched Tg1 (**S6C and S6D Fig;** *compare LD G2-depleted Tg1 to HD G2-depleted MA/My*, *and HD Tg1 spleen sections*). Despite that infected cell inclusion bodies were observed in both strains, Tg1 spleen was remarkable for increased granulocytosis and fibrinoid necrosis (**S6C and S6D Fig**). These results therefore demonstrate that *Cmv5*'s effect on SLO structural integrity during infection is independent of MCMV titers, and G2^+^ NK-mediated resistance. We infer that the combined effect of D^k^ and *Cmv5* ^*r*^ homozygosity in MA/My intensifies host resistance by protecting or regenerating spleen SLO structural features, preventing severe necrosis in spleen RP and promoting NK cell expansion needed to mediate specific virus clearance.

## Discussion

Although small-effect QTL were thought to increase the extent of D^k^-dependent resistance to MCMV in C57L, compared to MA/My background mice [26], until now they have eluded detection likely due to the robust effect of D^k^. A genome-driven integrated approach with multiparametric flow cytometry revealed 56 novel immune and MCMV responsive QTL. Though improbable that any two separately measured traits (e.g. splenic MCMV level and percentage G2^+^ NK cells postinfection) would map similarly, unless controlled by the same, or another closely linked gene, the combined approach helped to distinguish significant genomic linkages. Importantly, the combined genome scanning approach resulted in the discovery and mapping of multiple QTL, which together shape strain-specific variances associated with MCMV infection.

Consistent with its role in licensing G2^+^ NK cells [25,36], we observed that the H-2D locus regulated both naïve G2^+^ NK cell percentages and G2 receptor expression without specific effects on features in other NK cell subsets. H-2D also controlled G2^+^ NK cell percentages, and the severity of weight loss after MCMV exposure. Together the findings confirm that much of the genetic impact on host resistance is through D^k^-specific regulation of G2^+^ NK cells. Intriguingly, an interactive NKC-linked QTL further shaped MHC I D genetic regulation of G2^+^ NK cells since G2^c57l^ and G2^mamy^ receptors were expressed differently on NK cells in non-D^k^ mice. Lower G2^c57l^ receptor expression on a smaller percentage of NK cells is suggestive that these NK cells could be licensed, though not confirmed by *ex vivo* stimulation experiments. As the majority of NK cells express the given G2 receptor allele in C57L-derived NKC^m^ or NKC^l^ congenic mice, further analysis is needed to exclude potential effects due to other common licensing receptors. Moreover, whereas increased host resistance due to NKC^l^ further suggests that G2 receptor polymorphism might affect MCMV clearance differences, further analysis is needed to precisely define the role of G2^+^ NK cells. In aggregate, these findings suggest that chr-6 holds two or more QTL: one or more in the NKC that influence NK cell traits, and another more proximal locus, reported in Table 2, that regulates postinfection body weight. Therefore, the best interpretation is that an additive chr-6 effect with H-2D on MCMV resistance requires a more proximal locus, possibly in addition to a NKC-linked QTL.

Given H-2D’s profound influence over NK cells, it was not surprising that overall postinfection percentages of NKp46^+^ NK cells were controlled by a chr-17 QTL. The newly discovered *Cmv5* locus, however, segregated away from H-2D in both the LD and HD analyses. Two-dimensional genome scans of MCMV resistance and morbidity indices validated that at least one non-MHC chr-17 iQTL added to the genetic effect of H-2D. Though mapped proximally in the LD cohort such that a *Cmv5* CI spans the H-2D locus, we favor a more distal map location as obtained in the HD analysis, for several reasons. First, in addition to its larger size, the HD cohort included two different MHC congenic blocks (R7 and M.H2^b^), which increased both resolution (smaller CIs) and precision (more informative crossovers) in the genetic linkage analysis. Second, less morbidity amongst LD mice also might have limited the map analysis as *Cmv5’s* effect was most clear after HD-infection. Lastly, analysis of MA/My-derived congenic strains verified that *Cmv5* is distinct from and resides distal to H-2D on chr-17.

In addition to its effect on NK cells in infected spleen, we found that *Cmv5* also regulates spleen size, SLO structure and organization, and red pulp necrosis, which is at least partly via its direct effect in spleen and independent of G2^+^ NK cells. One possibility is that the *Cmv5* ^*r*^ resistance allele protects against the loss of splenic organization and other changes by increasing NK-mediated control of viral replication. An intrinsic modifier of cell proliferation or effector function, for example, might facilitate a more rapid NK response and sensing of viral targets. In fact, m157-specific Ly49H^+^ NK cells were recently shown to protect lymphoid organization in spleen red and white pulp within days after MCMV infection [35]. Extrinsic cues (i.e. cytokines or growth factors), on the other hand, could also aid in accrual of NK cells via increased recruitment, retention or enhanced proliferation in infected spleen tissue. *Cmv5* protection of, or enhancement of IL-15 production by, CD8α DC [37–39] or VCAM-1^+^ CD31^-^ stromal cells in spleen [40] might explain increased NK accumulation. FMS-like tyrosine kinase 3 ligand (Flt3-L), another interesting candidate, is needed to generate pDC and resident DC from Lin^-^Flt3^+^ bone marrow precursors [41], which subsequently stimulates DC to increase NK cell activation after MCMV infection [42]. Alternatively, *Cmv5*’s action may be directed to support specific spleen cell function(s) independent of NK-mediated viral control, especially given that resistance in Tg1 surpasses that in M.H2^b^ mice after LD- or HD-infection, without affecting other *Cmv5* features. Thus, D^k^-dependent MCMV resistance *per se* is inadequate to guard against virus-induced loss of splenic architecture. Whether a cell intrinsic effect or not, greater numbers of competent NK cells in the spleen that can limit viral spread would be expected to minimize excessive inflammation and tissue injury [43].

Recently, we performed whole exome sequencing of MA/My, C57L and M.H2^b^ to elucidate potential genetic candidates for *Cmv5*’s effect (H. Lee, A.G. and M.G.B., manuscript in preparation). In the critical genetic interval (~17-Mb) flanked by H2*D* and the M.H2^b^ congenic recombination crossover marked by *Sgol1*, 117 expressed sequences were assessed, including many with non-synonymous or frame shift variants that distinguish MA/My and C57L alleles. Notable among immune-related positional candidates are *Tnfrsf21*, and *Pla2g7* implicated in influenza control [44], though its effect was limited to male mice. The Trem /Trem-like gene cluster [45] also maps nearby *Cmv5*'s peak position. As these molecules have been shown to regulate innate immune cells [45], inflammation [46,47], dead cell clearance [48,49] and disease [50–53], they too represent interesting positional candidates.

During recovery from viral infection, the spleen and other organs can serve as sites of blood formation or extramedullary hematopoiesis (EMH) [54]. Indeed, spleen NK cells were also shown to promote effective EMH by specifically controlling viral spread [55]. It will be important to determine whether *Cmv5* regulates EMH, either apart from or in unison with D^k^-dependent NK-mediated MCMV resistance. Thus, this genetic model may extend beyond investigating the effect of MHC polymorphism on NK-mediated virus resistance to examine EMH regulation generally, and during viral infection.

Genetic regulation of splenic NK cell accumulation might lend additional precision to the host immune response. Whether due to increased splenic recruitment, retention, or enhanced proliferation that result from intrinsic or extrinsic effects, the fewer numbers of NK cells in the spleen of mice that carry a C57L-derived *Cmv5* ^*s*^ susceptibility allele should likewise decrease NK-mediated virus resistance. Alterations in NK numbers, and possibly activation status, have the potential to influence other immune cells in the response to infection. Specific NK-mediated control of MCMV has been shown to protect CD8α DC, which reciprocally affect the expansion of NK cells after infection [37], and the balance of NK-DC crosstalk has considerable potential to affect T cell immunity [56,57]. During persistent LCMV infection, NK cells were also shown to regulate adaptive immunity through direct lysis of CD8^+^ and CD4^+^ T cell effectors [58,59], which is subject to type I IFN regulation of MHC I expression on T cells themselves [60,61]. Likewise in MCMV infection, too few NK cells or inefficient NK activity may result in over exaggerated effector T-cell function, while too many NK cells can also harm otherwise protective T cell immunity [62,63]. Intriguingly, this effect of NK cells extends to humoral immunity, as follicular helper T cells are also a target of NK-mediated lysis after LCMV and other viral infections [64,65]. Thus, *Cmv5* regulation of the innate immune response to viral infection is an additional pivotal mechanism governing NK cell numbers and consequently, their impact on other critical immune cells in the host response, inflammation and tissue injury.

## Materials and Methods

### Ethics statement

All animal experiments conducted in this study were carried out in accordance with the Animal Welfare Act and the recommendations in the Guide for the Care and Use of Laboratory Animals of the National Institutes of Health. All experiments were approved by the University of Virginia Animal Care and Use Committee (Protocol Number: #3050).

### Mice

MA/My x C57L crossed offspring given HD MCMV were described previously [23]. Congenic blocks were included in the crosses to aid identification of small-effect, and possibly MHC- or NKC-linked QTL. Crossed mice without congenic blocks were also generated and analyzed following LD MCMV infection. A diagram of the breeding strategy is shown in **S1 Fig**. MA/My-derived MHC congenic M.*H2* ^*b*^ (i.e. MA/My.L-*H2* ^*b*^) and M.*H2* ^*b*^-TgD^k^ (Tg1) were described before [25,26]. Tg1 mice have been backcrossed to M.H2^b^ mice for 10+ generations, with marker-assisted genetic selection in the first 4–5 generations. Mice used in the study were managed with a Colony Management System (Jackson Labs, Version 4.1.2), maintained in a dedicated animal care facility under SPF conditions and treated in accordance with IACUC regulations and guidelines.

### *A*ntibodies, flow cytometry and immunofluorescence

Abs and isotype controls for flow cytometry were purchased from BD Biosciences, Biolegend, and R&D Systems. They included anti-CD16/32 (2.4G2), Ly49G2 (4D11 and AT8), Ly49D (4E5), Ly49A (YE1/48.10.6), Ly49I/U (14B11), CD3 (145-2C11), CD19 (6D5) and NKp46 (29A1.4). Abs were conjugated to FITC, PE, PerCP-Cy5.5, APC, APC-Cy7 or biotin followed by streptavidin conjugated to APC-Cy7 (Biolegend). Leukocyte stains, dead cell exclusion, compensation and cytometric analyses were performed as described [23,36]. To determine the licensing status and functional cytokine production by NK cells, *ex vivo* stimulation and intracellular cytokine staining were performed as previously described [66].

For IF staining of leukocytes and MZ, spleens were snap frozen in liquid nitrogen, processed at the UVA Research Histology Core and examined as described [67]. Briefly, frozen sections (6 μm) were fixed with a 1:1 mix of acetone/ethanol(100%) and stained with Abs against B220 (RA3-632, BD Bioscience), CD3 (145-2C11, Ebiosciences), MAdCAM-1 (MECA-367, BioXcell), CD209b/SIGN-R1 (eBio22D1, Ebiosciences), NKp46 (polyclonal, R&D systems), and CD169 (MOMA-1, AbDSerotec). Non-specific staining and endogenous peroxidase activity were blocked using the Avidin/Biotin blocking kit (Vector Labs), and 3%H2O2 + 10mM NaN3 solution, respectively. The reaction was amplified using the tyramide signal amplification technique (Perkin Elmer, Boston MA). Slides were incubated with primary Abs (1h), followed by secondary biotinylated or FITC-conjugated Abs (Vector Labs). Slides were then incubated with streptavidin-conjugated horseradish peroxidase and with biotinylated or FITC conjugated tyramide. Finally, streptavidin conjugated with Texas Red (Southern Tech) was used to visualize the reaction. Sections in which the primary Ab was omitted served as negative control. Sections were counterstained with DAPI.

### Histology

Spleens fixed with 10% phosphate-buffered formaldehyde were processed and stained at the UVA Research Histology Core and then examined as described [67].

### MCMV infection and quantification

Mice given HD (2 x 10^5^ PFU) MCMV were described previously [23]. Additional crossed mice were infected with LD (1 x 10^4^ PFU) MCMV and analyzed as described before [23]. Spleen and liver fragments were processed for genomic (g)DNA genotyping and quantitative real-time PCR (qPCR) analysis of MCMV genome level as described [23,68]. MA/My, M.H2^b^ and M.H2^b^-TgD^k^ received HD or LD MCMV and tissues were analyzed 3-7d after infection.

### Genotyping and quantitative trait analysis

Spleen and liver gDNA samples were prepared using a Gentraprep kit. A DNA 'footprint' of MHC, NKC and chromosome 19 genotypes was obtained for each mouse using gene-specific PCR strategies as described [23,32,69]. High-resolution melt PCR for H-2*D* exons 5 and 8 was used to validate cross and congenic mouse genotypes.

Genome-wide genotyping was performed at DartMouse (Dartmouth, NH) using an Illumina medium density SNP panel. Mouse genotypes were concatenated and transposed in Excel. Genomic profiles were reviewed for marker alignment and reliability. Those with greater than 20% failure rate (double recombination or non-informative) were discarded. Genomic profiles were then merged with quantitative trait data and stored in a Csv file.

LD- and HD-infected mice were studied and analyzed in two separate cohorts. QTL mapping was performed using R/qtl in the statistical computing program R [28]. Csv files were imported into R using read.cross. Calc.genoprob (5 cM step) was used to predict genotypes between markers. Each trait was analyzed using a One QTL scan (Haley-Knott method). QTL significance thresholds were calculated based on 10,000 permutations. QTL effect plots were generated using effectplot after running sim.geno (1 cM step) and n.draws (= 64) to obtain imputations. In some cases, the phenotype x genotype command was run to view phenotype values for individual animals at a given marker. Traits analyzed with two-dimensional genome scans (scantwo) in R/qtl had significance thresholds based on 1000 permutations.

### Data validation and statistical analysis

Assessment of data integrity for HD mice has been described [23]. Similar methods were used to assess and validate LD results. Statistical analyses included paired and unpaired Student T tests, Pearson correlations and multiple linear regression tests performed in R [70] with select plots drawn using the ggplot2 package [71]. Results obtained for MA/My-derived congenic strains were analyzed using one-way ANOVA in conjunction with Tukey’s test using Prism software (version 6.0d, 2013).

## Supporting Information

S1 Fig Schematic diagrams of the genetic crosses and flow charts depicting the genome-driven integrated analysis of the NK response to acute viral infection. A) The diagram depicts the genetic crosses used to generate offspring for inclusion in the LD and HD cohorts under study. B) The diagram depicts the procedures used to evaluate naïve and infected animals, tissues, immune cells and genome-wide genotypes in the integrated genomic analysis, including multiparametric flow cytometric analysis of pre- and postinfection immune cells. C) A flow-through diagram of the procedure used to prepare and then analyze independently measured traits for each cohort using one- and two-dimensional genome scans in R/qtl.
(TIF)

S2 Fig Multiple NK cell and host response QTL together enhance host resistance to MCMV. (A) The chromosome maps depict QTL positions associated with MCMV immunity that were detected in genome scans of experimental traits reported in **Table 1**, and validated in **Table 2**. A relative LOD value range for black (3.8 ≤ LOD < 6), blue (6 ≤ LOD < 16), and red (16 ≤ LOD) QTL positions on the genome-wide map is represented. (B) The heat map depicts the type (epistatic or additive), magnitude (based on LOD_int_ or LOD_av1_ values, respectively) and predicted position for each significant HD QTL effect / interaction for the indicated experimental traits.
(TIF)

S3 Fig C57L-derived NKC^l^ and NKC^m^ NK cells display comparable responsiveness to activation receptor stimulation. The plots show licensing ratios for NK cells from the indicated strains following stimulation with plate-bound PK136 mAb as described previously [25,66]. Results are representative of two independent experiments.
(TIFF)

S4 Fig G2^+^ NK cell responsiveness in non-D^k^-expressing C57L mice corresponds to increased MCMV resistance in NKC-disparate, MHC-matched M.H2^b^ mice. (A) Licensing ratios are shown for G2^+^ NK cells in M.H2^b^ and C57L stimulated with plate-bound mAbs to activating receptors, NK1.1 or NKp46. (B and D) The plots show naïve peripheral blood (B) and LD-infected (d4) spleen (D) G2^+^ NK cell features. (C) The plot shows MCMV genome levels (d4) for individual M.H2^b^ and C57L spleens. (E-G) The plots represent numbers per mg spleen and frequencies of IFN-γ+ NK cells (E) and IFN-γ+ G2^+^ NK cells (F), in addition to IFN-γ gMFI values (G) for both total and G2^+^ NK cells. Statistics were performed using an unpaired Student’s t-test (*p < .05, **p < .01, ***p < .001).
(TIF)

S5 Fig NK1.1+ and NKp46+ NK cells display similar expansion after MCMV infection in MA/My mice. The plots show percentages of NK1.1^+^ and NKp46^+^ NK cells in HD-infected MA/My, M.H2^b^ and Tg1 (M.H2^b^ background) mice.
(TIF)

S6 Fig *Cmv5* imparts protection of splenic SLO structures and lymphoid remodeling independent of G2^+^ NK cells and viral control. (A) Spleen weights (left) and total splenocytes recovered (right) are plotted for LD- and HD-infected (d4) MA/My and Tg1 mice treated with either rat isotype IgG (rIgG) or G2-depleting mAb 4D11. (B) The total number (left) and frequency (right) of NKp46^+^ NK cells in LD- and HD-infected (d4) spleens are shown. (C) The plot shows MCMV genome levels in LD- and HD-infected MA/My and Tg1 (±mAb 4D11 treatment) spleens. (D) Representative H&E-stained spleen sections for LD- and HD-infected (d4) MA/My and Tg1 mice with the indicated Ab treatment are shown (magnification X100). Images are representative of 4 mice per group and per dose. Irregularities in the structure, content, and dominance of WP regions are evident in different mouse strains and across viral doses. In addition to the increased dominance of RP observed in Tg1 mice, greater degrees of fibrinoid necrosis, granulocytosis, and viral particle presence are noted. Statistics were calculated using two-way ANOVA in conjunction with Sidak’s test (*p < .05, **p < .01, ***p < .001, ****p < .0001).
(TIF)
