# Supplementary figures and images for: Genomic Modifiers of Natural Killer Cells, Immune Responsiveness and Lymphoid Tissue Remodeling Together Increase Host Resistance to Viral Infection
Source: PLoS Pathog. 2016 Feb 4;12(2):e1005419. doi: 10.1371/journal.ppat.1005419 (PMC4742223; doi:10.1371/journal.ppat.1005419)

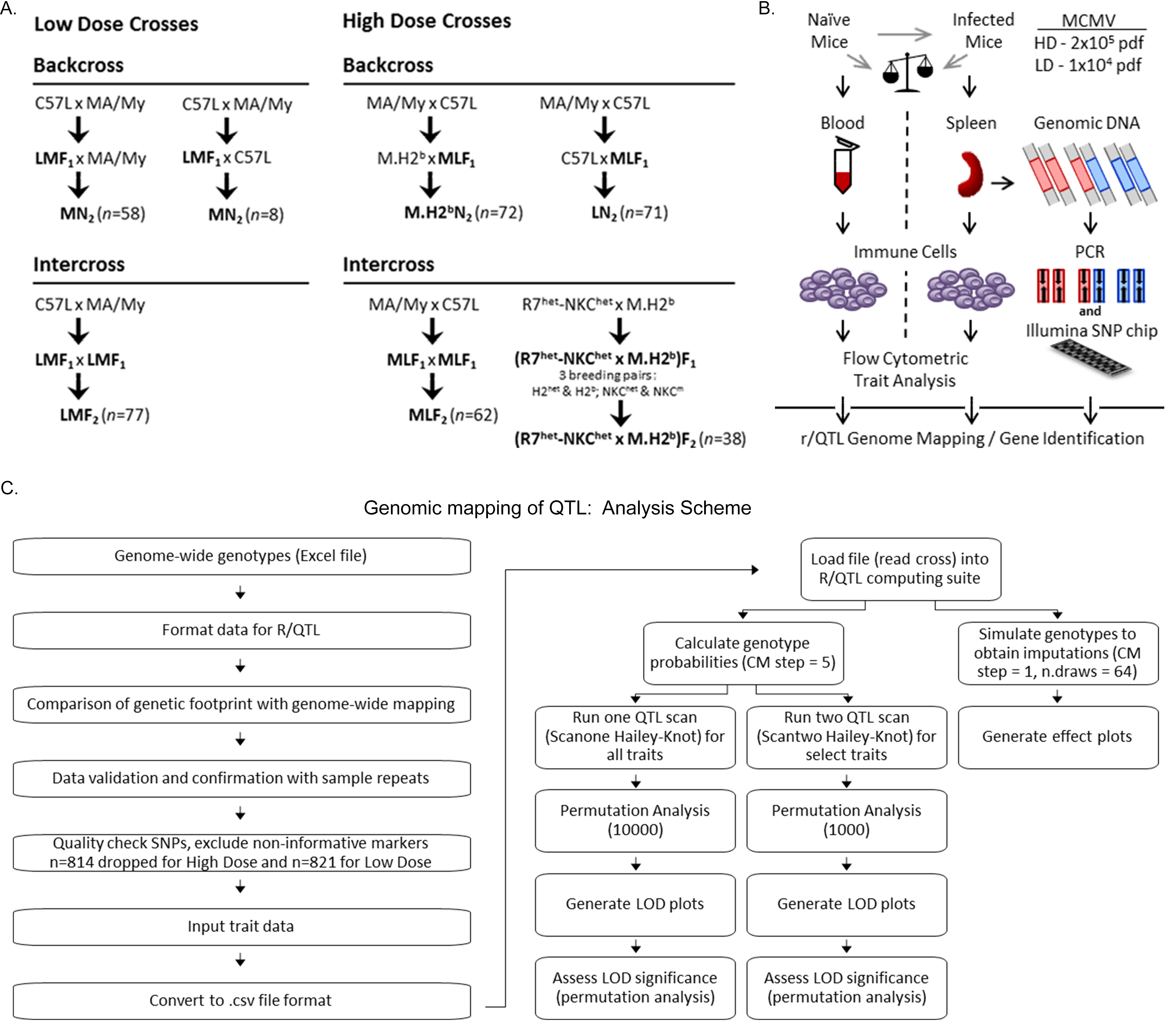

Supplement: S1 Fig — A) The diagram depicts the genetic crosses used to generate offspring for inclusion in the LD and HD cohorts under study. B) The diagram depicts the procedures used to evaluate naïve and infected animals, tissues, immune cells and genome-wide genotypes in the integrated genomic analysis, including multiparametric flow cytometric analysis of pre- and postinfection immune cells. C) A flow-through diagram of the procedure used to prepare and then analyze independently measured traits for each cohort using one- and two-dimensional genome scans in R/qtl. (TIF) [file ppat.1005419.s001.tif]

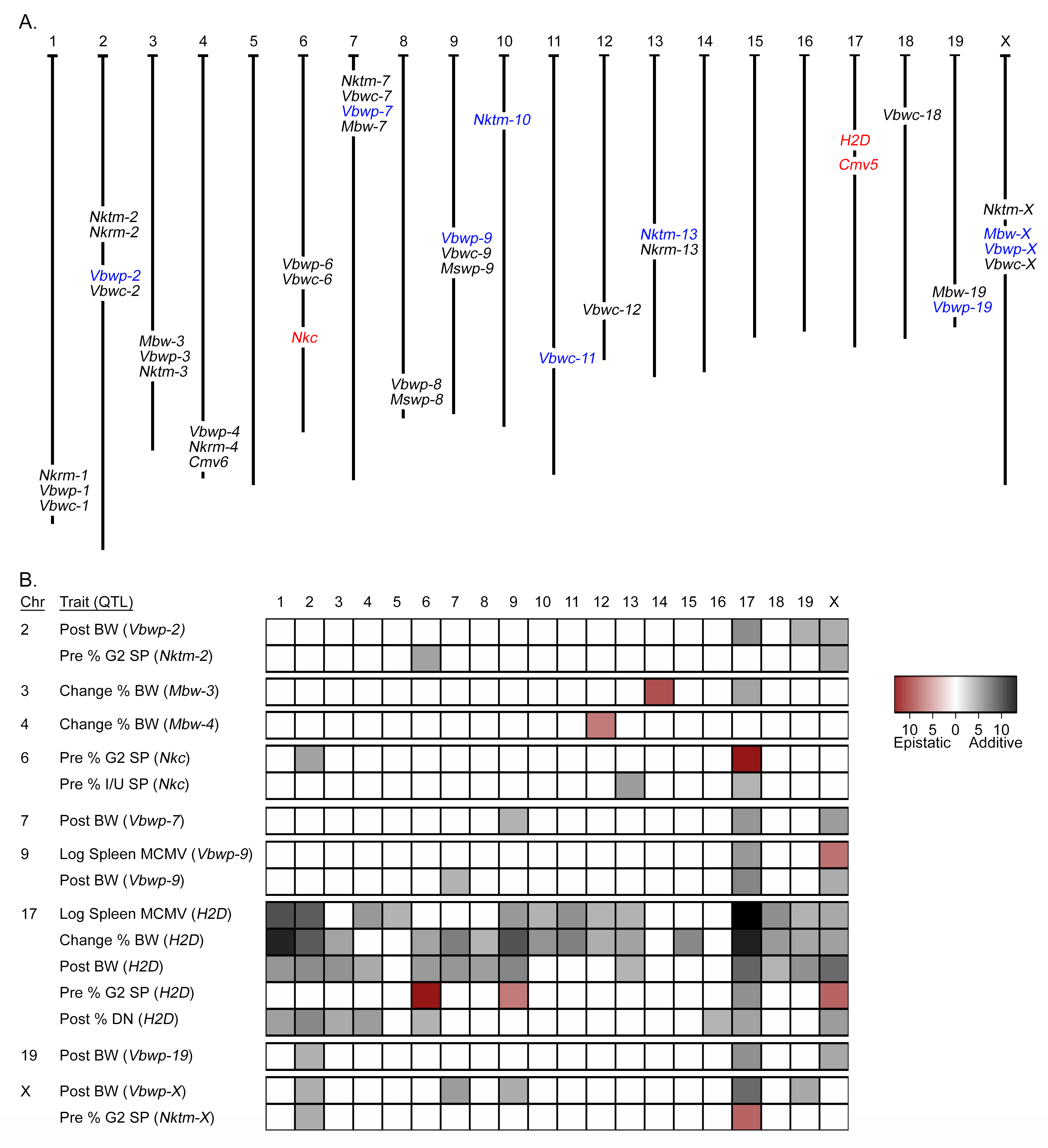

Supplement: S2 Fig — (A) The chromosome maps depict QTL positions associated with MCMV immunity that were detected in genome scans of experimental traits reported in Table 1, and validated in Table 2. A relative LOD value range for black (3.8 ≤ LOD < 6), blue (6 ≤ LOD < 16), and red (16 ≤ LOD) QTL positions on the genome-wide map is represented. (B) The heat map depicts the type (epistatic or additive), magnitude (based on LODint or LODav1 values, respectively) and predicted position for each significant HD QTL effect / interaction for the indicated experimental traits. (TIF) [file ppat.1005419.s002.tif]

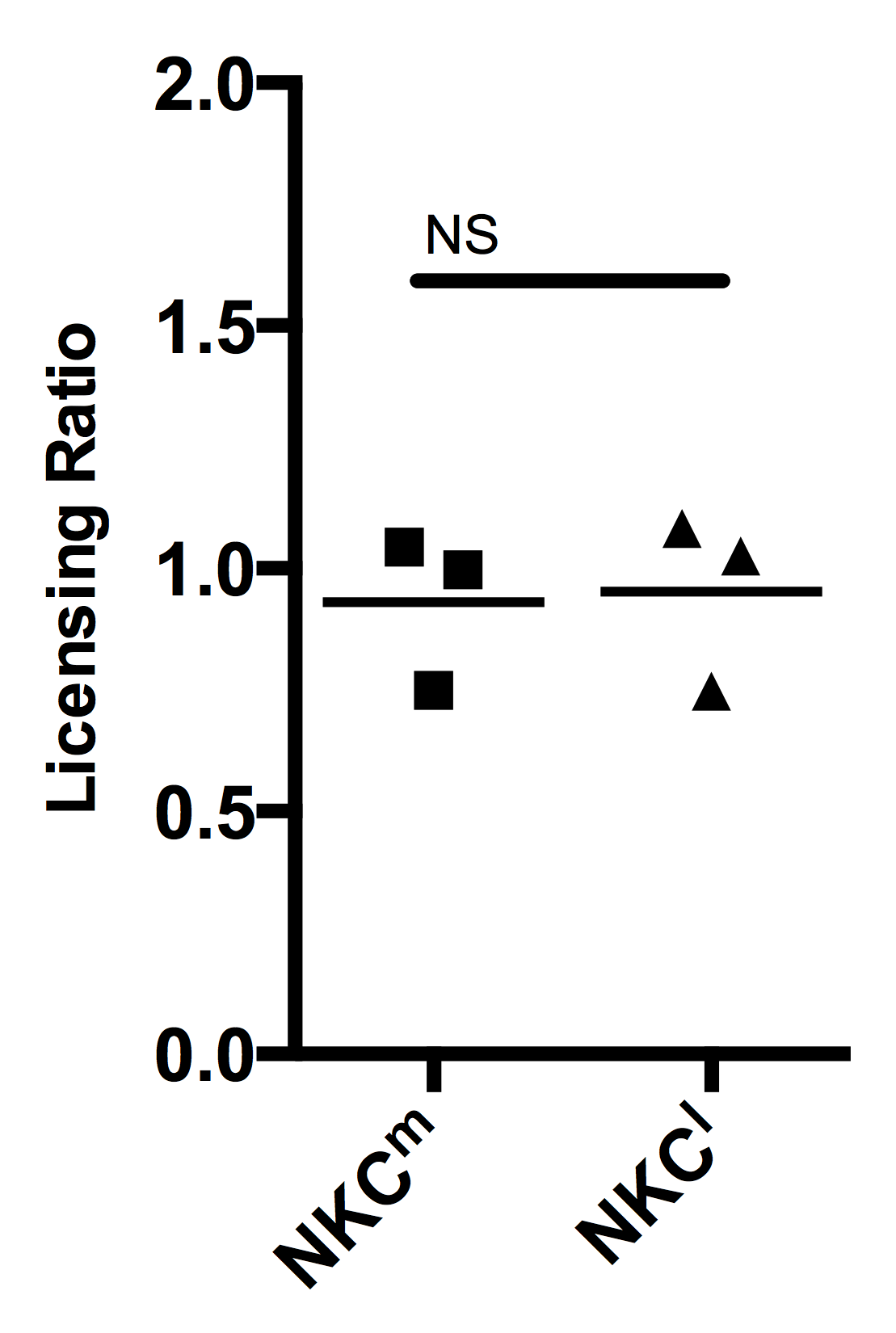

Supplement: S3 Fig — The plots show licensing ratios for NK cells from the indicated strains following stimulation with plate-bound PK136 mAb as described previously [25,66]. Results are representative of two independent experiments. (TIFF) [file ppat.1005419.s003.tiff]

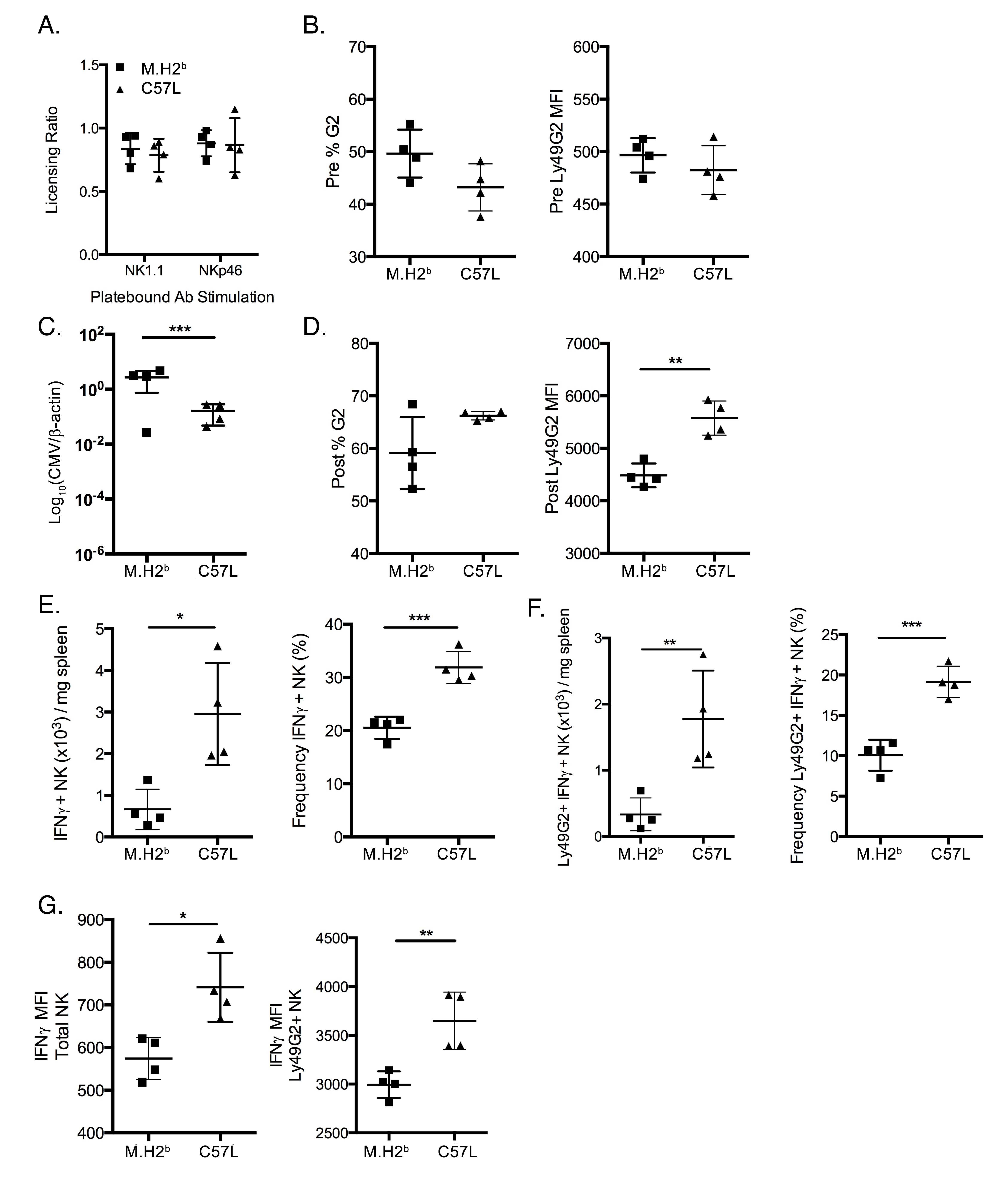

Supplement: S4 Fig — (A) Licensing ratios are shown for G2+ NK cells in M.H2b and C57L stimulated with plate-bound mAbs to activating receptors, NK1.1 or NKp46. (B and D) The plots show naïve peripheral blood (B) and LD-infected (d4) spleen (D) G2+ NK cell features. (C) The plot shows MCMV genome levels (d4) for individual M.H2b and C57L spleens. (E-G) The plots represent numbers per mg spleen and frequencies of IFN-γ+ NK cells (E) and IFN-γ+ G2+ NK cells (F), in addition to IFN-γ gMFI values (G) for both total and G2+ NK cells. Statistics were performed using an unpaired Student’s t-test (*p < .05, **p < .01, ***p < .001). (TIF) [file ppat.1005419.s004.tif]

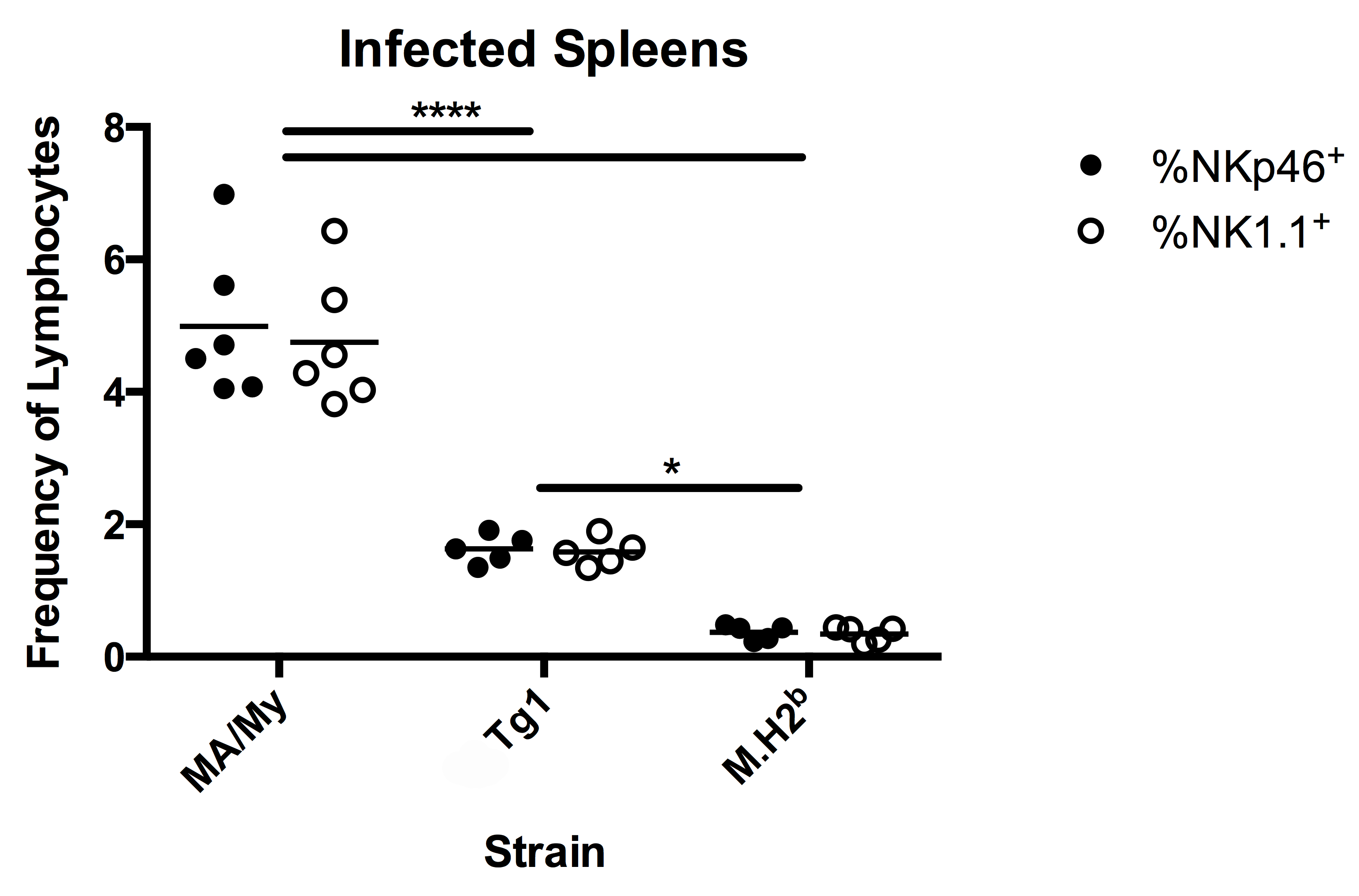

Supplement: S5 Fig — The plots show percentages of NK1.1+ and NKp46+ NK cells in HD-infected MA/My, M.H2b and Tg1 (M.H2b background) mice. (TIF) [file ppat.1005419.s005.tif]

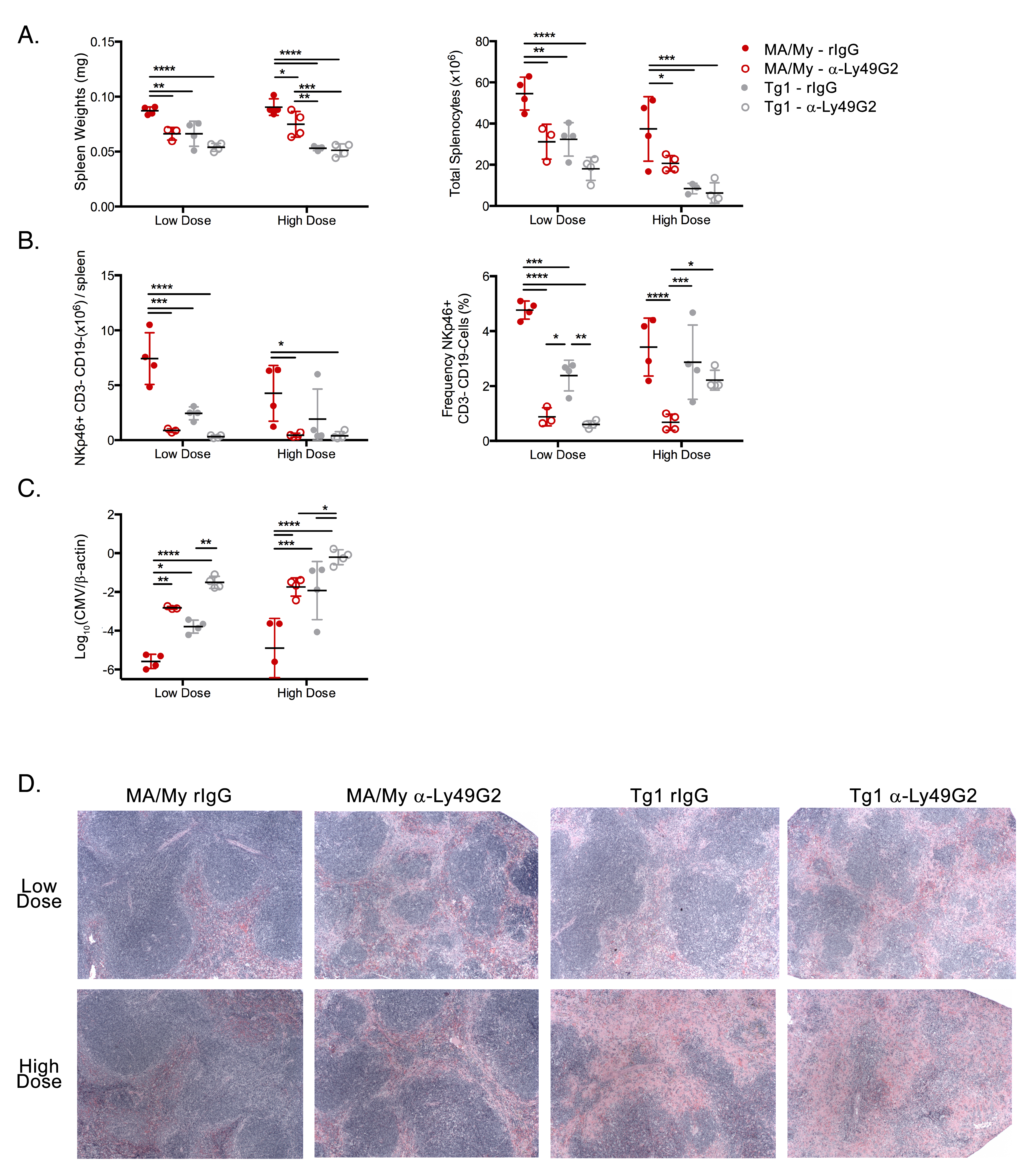

Supplement: S6 Fig — (A) Spleen weights (left) and total splenocytes recovered (right) are plotted for LD- and HD-infected (d4) MA/My and Tg1 mice treated with either rat isotype IgG (rIgG) or G2-depleting mAb 4D11. (B) The total number (left) and frequency (right) of NKp46+ NK cells in LD- and HD-infected (d4) spleens are shown. (C) The plot shows MCMV genome levels in LD- and HD-infected MA/My and Tg1 (±mAb 4D11 treatment) spleens. (D) Representative H&E-stained spleen sections for LD- and HD-infected (d4) MA/My and Tg1 mice with the indicated Ab treatment are shown (magnification X100). Images are representative of 4 mice per group and per dose. Irregularities in the structure, content, and dominance of WP regions are evident in different mouse strains and across viral doses. In addition to the increased dominance of RP observed in Tg1 mice, greater degrees of fibrinoid necrosis, granulocytosis, and viral particle presence are noted. Statistics were calculated using two-way ANOVA in conjunction with Sidak’s test (*p < .05, **p < .01, ***p < .001, ****p < .0001). (TIF) [file ppat.1005419.s006.tif]
